# Location-dependent synaptic plasticity rules by dendritic spine cooperativity

**DOI:** 10.1038/ncomms11380

**Published:** 2016-04-21

**Authors:** Jens P. Weber, Bertalan K. Andrásfalvy, Marina Polito, Ádám Magó, Balázs B. Ujfalussy, Judit K. Makara

**Affiliations:** 1 Momentum Laboratory of Neuronal Signaling, Institute of Experimental Medicine, Hungarian Academy of Sciences, 43 Szigony Street, Budapest 1083, Hungary

## Abstract

Nonlinear interactions between coactive synapses enable neurons to discriminate between spatiotemporal patterns of inputs. Using patterned postsynaptic stimulation by two-photon glutamate uncaging, here we investigate the sensitivity of synaptic Ca^2+^ signalling and long-term plasticity in individual spines to coincident activity of nearby synapses. We find a proximodistally increasing gradient of nonlinear NMDA receptor (NMDAR)-mediated amplification of spine Ca^2+^ signals by a few neighbouring coactive synapses along individual perisomatic dendrites. This synaptic cooperativity does not require dendritic spikes, but is correlated with dendritic Na^+^ spike propagation strength. Furthermore, we show that repetitive synchronous subthreshold activation of small spine clusters produces input specific, NMDAR-dependent cooperative long-term potentiation at distal but not proximal dendritic locations. The sensitive synaptic cooperativity at distal dendritic compartments shown here may promote the formation of functional synaptic clusters, which in turn can facilitate active dendritic processing and storage of information encoded in spatiotemporal synaptic activity patterns.

## Abstract (web summary)

Inputs to functionally related synapses have been suggested to show cooperative summation, although the rules governing these interactions are unclear. Here, Weber *et al*. uncover non-linear interactions dependent on NMDAR signalling that vary across the proximal-distal axis of individual dendrites.

## Graphical abstract

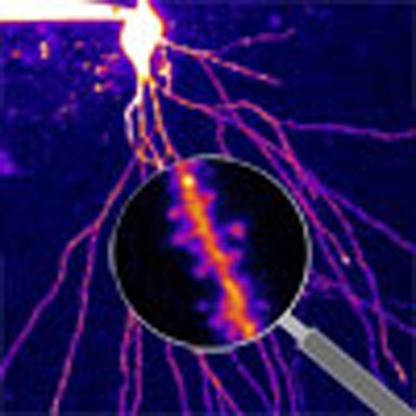

Most central principal neurons are constantly bombarded by excitatory synaptic inputs arriving onto spines in the dendritic tree. Distinct spatiotemporal patterns of inputs may be embedded in this activity, produced by the correlated activity of different presynaptic neuronal ensembles that code specific features of the environment. An essential question is whether such functionally related excitatory inputs are distributed in a random or a spatially clustered manner on the dendritic tree, and if the latter, what the underlying mechanisms implementing structured connectivity are. Recent data suggest that in hippocampal pyramidal cells (PCs) functionally related inputs arriving from CA3PCs can form small clusters on short segments of individual basal or oblique dendrites^1^,^2^,^3^,^4^, which may be consistent with the observations of hotspots of excitatory synaptic activity in hippocampal PC dendrites *in vivo*^5^,^6^. Furthermore, clustered potentiation or formation of synapses was revealed during sensory experience or learning in cortical PCs *in vivo*^7^,^8^,^9^,^10^. Since synaptic integration and plasticity are thought to depend on the number (classically termed ‘cooperativity') and spatiotemporal pattern of activated inputs^11^,^12^, this structured synaptic arrangement throws new light on fundamental questions about interaction and cooperation between small clusters of inputs. Is the function of a synapse sensitive to coincident activity in a single or a few adjacent synapses? What are the threshold activities for different types of nonlinear interactions among synapses and how do these thresholds depend on the precise spatiotemporal pattern and dendritic location of the inputs? Could such local interactions in synaptic function lead to long-term changes in synaptic efficacy?

Although dendritic spines, harbouring most of the excitatory synapses, can sequester Ca^2+^ and to some extent voltage signals evoked synaptically^13^,^14^,^15^,^16^,^17^,^18^, chemical and electrical crosstalk may overcome compartmentalization when active synapses are located close to each other. Since spines and dendrites are equipped with voltage-sensitive conductances such as NMDA receptors (NMDARs) and voltage-gated channels, coactive electrical and biochemical signals can be processed nonlinearly in both the compartments^17^,^18^,^19^,^20^,^21^,^22^,^23^,^24^,^25^,^26^,^27^,^28^,^29^,^30^,^31^,^32^. Several studies investigated cooperative interactions by various spatiotemporal patterns of multiple synaptic inputs, mainly focusing on the dendritic mechanisms producing supralinear integration of excitatory postsynaptic potentials (EPSPs)^20^,^21^,^22^,^23^,^24^,^25^,^26^,^27^,^28^,^29^,^30^,^31^,^32^. It is well documented that extensive synaptic activation in a dendritic region of hippocampal and neocortical PCs can reach the threshold of regenerative local dendritic spikes mediated by voltage-gated Na^+^ or Ca^2+^ channels (VGNCs, VGCCs), or NMDARs, depending on the dendritic region^31^. These events attracted much interest for providing supralinear dendritic signals influencing somatic action potential (AP) output and inducing synaptic plasticity^11^,^33^,^34^,^35^,^36^.

Interestingly, in most studies demonstrating the spatial clustering of functionally related synapses, the clusters were surprisingly small and tight, consisting of only a few synapses (approximately two to six) located on short dendritic segments (∼5–15 μm)^2^,^3^,^4^,^7^,^8^,^9^. Sporadic activation of such small clusters likely produces depolarization in the voltage range subthreshold for dendritic spikes, where EPSPs sum roughly linearly^20^,^22^,^37^. However, linear EPSP integration does not mean that the activated synapses do not interact. Because synaptic activity is translated to spine Ca^2+^ signals by NMDARs and VGCCs that are sensitive to depolarization, local spread of EPSPs from neighbouring synapses may cooperatively influence spine Ca^2+^ signals and downstream biochemical processes^38^ involved in the regulation of synaptic strength in activated spines, even in the locally subthreshold voltage range. The threshold requirements for cooperation of synaptic Ca^2+^ signals in nearby spines under physiological conditions are not well elucidated and may vary with neuron and synapse type^4^,^17^,^39^,^40^. Also, the mechanisms leading to such fine-scale clustered connectivity outcomes are largely unknown, as most plasticity experiments employ bulk electrical or optical stimulation techniques, where the number and localization of the activated synapses is not well controlled. Although cooperative forms of synaptic plasticity between adjacent synapses were suggested using two-photon glutamate uncaging (2PGU)^41^,^42^,^43^, their induction by different (electrically sub- and suprathreshold) spatiotemporal multisynaptic activity patterns has not yet been experimentally investigated.

Synaptic interactions are expected to depend on properties of the parent dendrite. In CA1PCs, most Schaffer collateral synapses are located on single or bifurcating families of basal and apical oblique dendrites with long (>40 μm), thin (diameter ∼0.3–0.7 μm) and tapering terminal branches^44^,^45^,^46^. Computer simulations indicate that the input impedance (which is low in the soma and trunk) increases drastically along thin dendrites from their origin to their tip^17^,^18^,^47^, markedly affecting the amplitude and kinetics of local dendritic depolarization by single synaptic inputs^17^,^18^,^47^. Accordingly, it is well documented that the properties of regenerative dendritic spikes depend on dendritic location of the activated inputs^22^,^26^,^27^,^28^,^29^. It may logically follow that location dependency may extend to subthreshold cooperative synaptic interactions^17^ and perhaps to plasticity, but this hypothesis has not yet been tested experimentally.

Here we used two-photon imaging (2PI) and 2PGU combined with somatic patch-clamp electrophysiology to examine the cooperative synaptic signalling by small input clusters. By imaging Ca^2+^ in a small number of coactivated spines on a short dendritic segment, we first show that the level of cooperativity of NMDAR-mediated spine Ca^2+^ signals, but not that of somatic summation of EPSPs, strongly depends on the dendritic location and spatiotemporal pattern of small, subthreshold input clusters. We next provide evidence that this subthreshold spine Ca^2+^ cooperativity is modulated by dendritic K^+^ channels and correlated with local dendritic Na^+^ spike strength. Finally, we demonstrate that repetitive synchronized activity of small spine clusters leads to input-specific cooperative long-term potentiation (LTP) of synaptic strength in a location-dependent manner that is consistent with the location dependence of cooperative Ca^2+^ signalling.

## Results

### Spatial gradient of cooperative spine Ca^2+^ signalling

**Figure 1:**
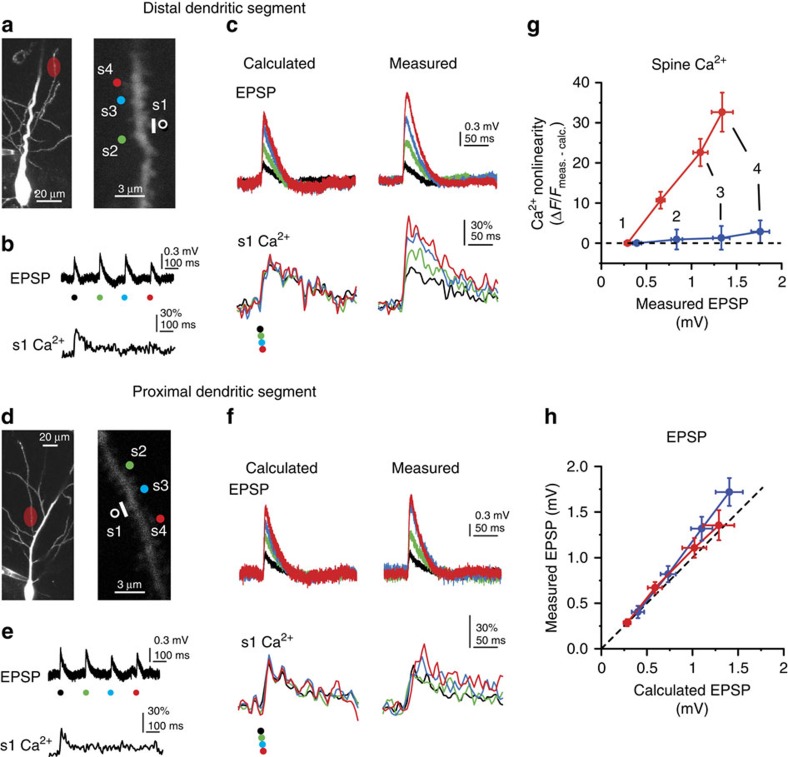
Cooperative spine head Ca^2+^ signalling in distal but not proximal dendritic segments. (**a**) Left: 2P *z*-stack of a CA1PC, with the uncaging location indicated in red at a distal site on an oblique dendrite. Right: magnified image of the stimulated segment. The four stimulated spines are indicated. (**b**) Representative recording of uncaging-evoked somatic EPSPs (upper trace) and Ca^2+^ signal in the first spine (lower trace) by individual stimulation of the four spines shown in **a**. (**c**) Calculated (left) and measured (right) somatic voltage traces (upper) and spine Ca^2+^ signals in s1 (lower) achieved by synchronous stimulation of the spines in increasing numbers (black: s1 alone; green: s1+s2; blue: s1+s2+s3; red: s1+s2+s3+s4). Note that, in the absence of interspine interactions, spine Ca^2+^ signals are expected to remain unaffected by stimulation of other inputs. **d**–**f** same as **a**–**c**, for spines stimulated at a proximal site on an oblique dendrite. (**g**) Quantification of cooperativity of synaptic Ca^2+^ signalling by calculating the nonlinear component of spine Ca^2+^ signals (measured minus calculated) at distal (red, *n*=18 spines, 6 cells, s1 and s2 data pooled) and proximal (blue, *n*=13 spines, 5 cells, s1 and s2 data pooled) dendritic segments, as a function of the somatically measured EPSP with increasing number of stimulated spines. (**h**) Measured versus calculated peak somatic EPSPs evoked at distal (red, *n*=10 experiments) and proximal (blue, *n*=7 experiments) dendritic segments with increasing number of stimulated spines. Group data are presented as mean±s.e.m. calc., calculated; meas., measured.

**Figure 2:**
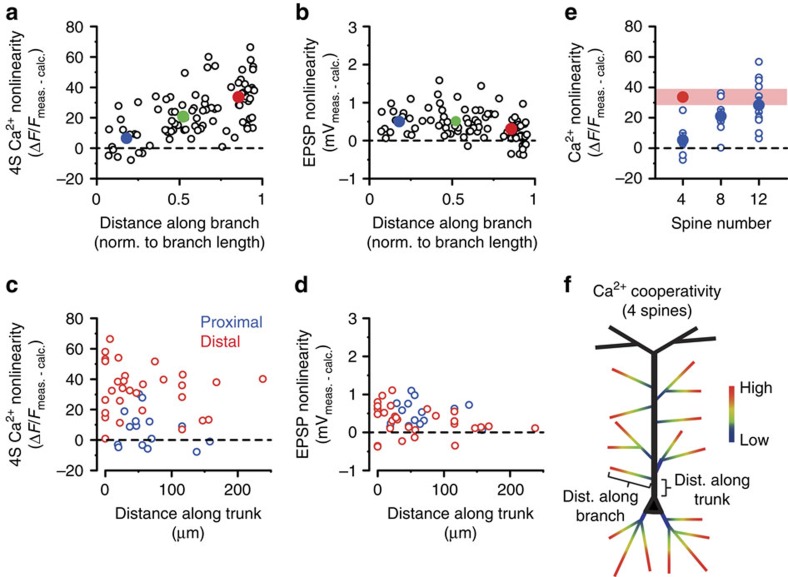
Dendritic map of synaptic cooperativity. (**a**,**b**) Cooperative spine Ca^2+^ nonlinearity (**a**, as described in Fig. 1g) and somatic EPSP nonlinearity (**b**, difference between measured and calculated peak amplitudes) evoked by four coactivated spines at different relative locations along individual branches. Open circles represent individual spine sets (results of all four spines averaged, 1 set/branch; middle branch data points are from experiments in Fig. 6). Filled symbols and error bars represent mean±s.e.m. for proximal (relative location (RL)<0.33, blue, *n*=16 experiments in 11 cells), middle (RL=0.33–0.67, green, *n*=33 experiments in 20 cells) and distal (RL>0.67, red, *n*=37 experiments in 25 cells) locations. Correlations: spine Ca^2+^ nonlinearity (**a**): Spearman *R*=0.606, *P*<0.001; somatic EPSP nonlinearity (**b**): Spearman *R*=−0.379, *P*<0.001. (**c**,**d**) Cooperative spine Ca^2+^ nonlinearity (**c**) and somatic EPSP nonlinearity (**d**), evoked by four coactivated spines located proximally (blue) or distally (red) within apical oblique dendrites, as a function of the distance of the originating branch point from the soma. Spearman rank correlations; (**c**) proximal: *R*=−0.193, *P*=0.490, *n*=15; distal: *R*=−0.097, *P*=0.608, *n*=30; (**d**) proximal: *R*=0.044, *P*=0.874; distal: *R*=−0.370, *P*=0.044. (**e**) Spine Ca^2+^ nonlinearity using increasing number of inputs in proximal dendritic segments (blue, *n*=6/7/11 for clusters of 4, 8 and 12 spines, respectively). Red symbol and band represent mean and 95% confidence interval, respectively, of the data obtained in distal segments with four spines. Comparison of 12S proximal and 4S distal data: Mann–Whitney test, *P*=0.404. (**f**) Schematics of the dendritic cooperativity map and distance measurements. dist., distance; calc., calculated; meas., measured; norm., normalized.

To quantitatively investigate synaptic interactions in small clusters of inputs, we exploited the spatiotemporal precision of 2PGU to stimulate spines in CA1PCs in acute slices from adult rats. We measured somatic voltage and spine Ca^2+^ responses to increasing numbers (up to four on an approximately 3–6 μm dendritic segment) of individually or synchronously stimulated nearby synapses, and compared measured signals with that expected from independent (arithmetically summing) synapses. Considering potential location-dependent differences of synaptic interactions due to variable passive dendritic properties, we performed our initial experiments at the two extremes of the branch impedance gradient, that is, at proximal (relative distance: 21±3% of branch length, *n*=7) versus distal (relative distance: 93±1%, *n*=10) dendritic locations along thin apical oblique and basal dendrites (Fig. 1a,d). We first stimulated four synapses individually (200–305 ms interspine stimulus interval, ISI), adjusting the laser power to yield uncaging-evoked EPSPs at each spine with somatic amplitudes similar to that of miniature EPSP evoked at different dendritic locations by local puffing of high-osmolarity artificial cerebrospinal fluid (ACSF; Supplementary Fig. 1a–f). To reduce contamination from spontaneous synaptic activity and to better assess the impact of passive dendritic properties, in most experiments, 0.5–1 μM tetrodotoxin (TTX) was included in the bath solution, which eliminates somatic and dendritic Na^+^ spikes^22^ and other potential nonlinearities of spine Ca^2+^ signalling^19^ (similar results were found without TTX, see below). EPSPs were usually accompanied by NMDAR-mediated spine head Ca^2+^ signals (Fig. 1b,e; Supplementary Fig. 1g) similar to those evoked by axonal stimulation^48^. Synaptic Ca^2+^ signals were mostly restricted to the activated spine and had similar amplitudes at proximal and distal dendritic locations (Fig. 1b,e; Supplementary Fig. 2a,b,g). Next, the same spines were stimulated in increasing number synchronously (0.1 ms ISI; see Methods). At distal dendritic sites, this stimulation produced pronounced amplification of spine Ca^2+^ signals, with a nonlinear increase of spine Ca^2+^ signal amplitude with activation of each additional nearby spine (Fig. 1c,g; Supplementary Fig. 2a,c; see also Fig. 2a and Supplementary Fig. 2f for larger data set with four and two spines). Coactivation of all four spines almost doubled the fluorescent signal in the activated spines (Supplementary Fig. 2a), accompanied by increased dendritic Ca^2+^ levels near the input site (Supplementary Fig. 2d). In contrast to distal dendritic locations, little if any nonlinearity of spine Ca^2+^ signals was detected at proximal locations using an identical stimulation protocol (Fig. 1d–g; Supplementary Fig. 2; two-way repeated measures analysis of variance (ANOVA): interaction between spine *N* and location: *P*<0.01, effect of location: *P*<0.001, effect of spine *N*: *P*<0.001). The strong cooperativity at distal (but not proximal) dendritic locations could not be explained by the location-dependent differences in dye-loading or dialysis (Supplementary Fig. 2j) nor by the extracellular glutamate diffusion (Supplementary Fig. 3a–f). In contrast to spine Ca^2+^ signals, somatically measured integration of EPSPs was largely linear with a slight gain at both proximal and distal locations (Fig. 1c,f,h), consistent with previous studies^22^,^37^.

We next sought to more precisely map the dendritic location rules determining synaptic Ca^2+^ cooperativity. Measuring nonlinearity of spine Ca^2+^ and somatic voltage responses to separate versus synchronous activation of four nearby spines (termed 4S condition) at various relative distances along basal and apical oblique thin dendrites revealed a clear proximodistal gradient of cooperative amplification of spine Ca^2+^ signals (Fig. 2a; a lower-affinity Ca^2+^ indicator yielded similar results (Supplementary Fig. 4a,b)). In contrast, the position of the branch in stratum radiatum and oriens did not matter: results were similar in basal and apical oblique dendrites (Supplementary Fig. 2k), and, for apical obliques, the distance of their originating branch point along the trunk from the soma did not correlate with 4S Ca^2+^ cooperativity at either proximal or distal (that is, along the oblique branch) input sites (Fig. 2c). In contrast to Ca^2+^ cooperativity, the small EPSP nonlinearity slightly decreased with distance along dendrites towards the tip (Fig. 2b), and for distal input sites, slightly correlated negatively with distance of the originating branch point along the trunk (Fig. 2d). Although only small Ca^2+^ nonlinearity was detected with the 4S protocol at proximal segments, ∼12 clustered proximal inputs were sufficient to produce similar Ca^2+^ nonlinearity as that measured with four inputs at distal sites (Fig. 2e). In summary, the threshold sensitivity of synaptic Ca^2+^ cooperativity increases gradually along thin dendrites from their base (∼16% supralinearity in fluorescence) to their tip (∼80% supralinearity) systematically in the dendritic target area of Schaffer collaterals (Fig. 2f), a pattern well matching the passive impedance profile of the dendritic arbour^17^. In contrast, the corresponding EPSPs sum at the soma largely linearly with little location dependence.

### Mechanism of cooperative spine Ca^2+^ signalling

**Figure 3:**
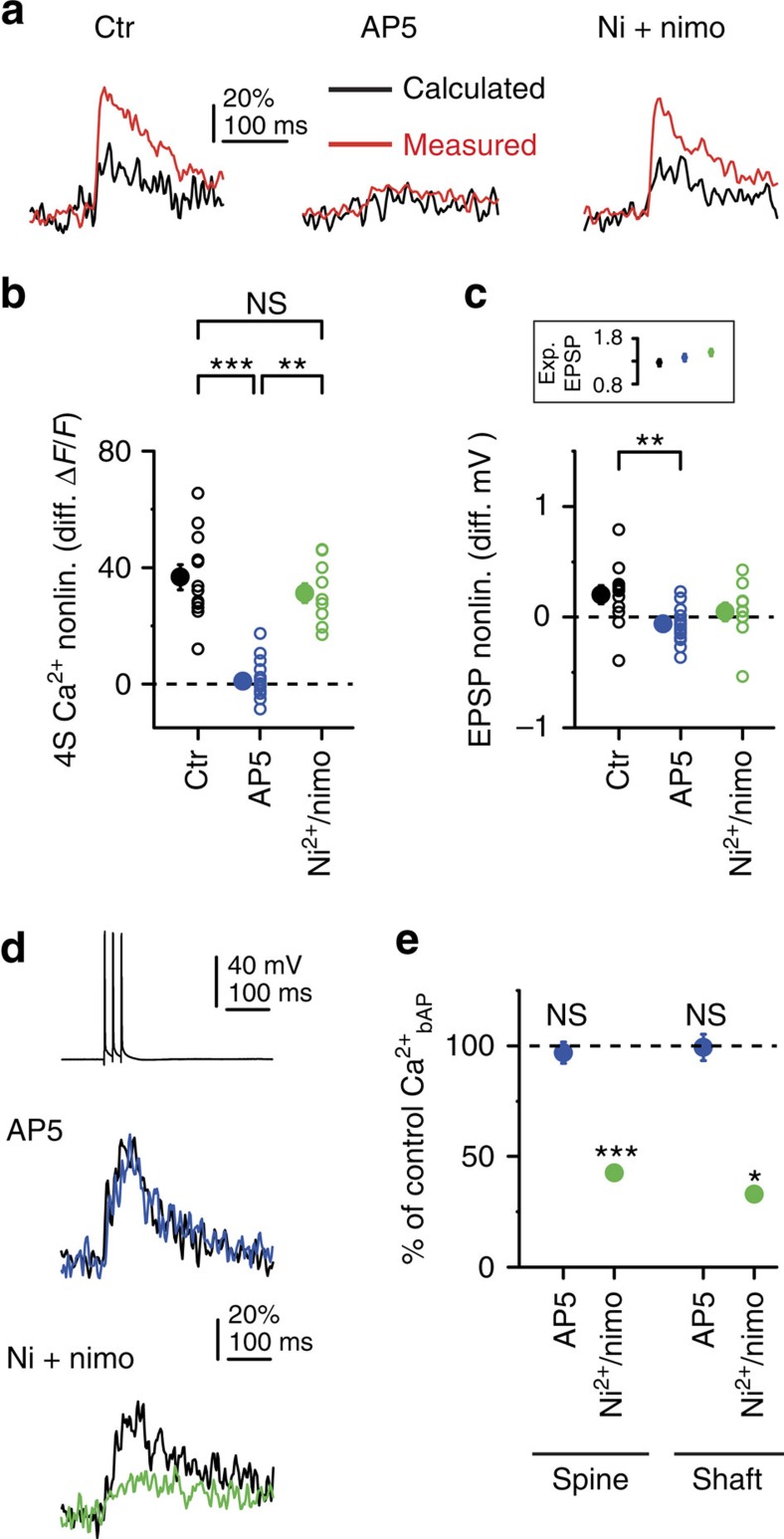
Cooperative spine Ca^2+^ signalling at distal dendritic locations is mediated by NMDARs. (**a**) Calculated and measured spine Ca^2+^ signals from representative experiments using the 4S protocol (averaged data from all four spines shown in each case) at distal segments of oblique and basal dendrites under control conditions (left), in the presence of AP5 (100 μM, middle) or in the presence of Ni^2+^ (100 μM) and nimodipine (20 μM) (right). (**b**,**c**) Summary of cooperative spine Ca^2+^ nonlinearity (**b**, multiple comparisons after Kruskal–Wallis test with *P<*0.001) and somatic EPSP nonlinearity (**c**, multiple comparisons after Kruskal–Wallis test with *P*<0.01) measured with the 4S protocol under control conditions (black, *n*=12 in 12 dendrites, five cells), in the presence of AP5 (blue, *n*=16 in 10 dendrites, three cells) and in the presence of Ni^2+^ and nimodipine (green, *n*=10 in 10 dendrites, four cells). Inset in **c** shows similar calculated EPSPs under all conditions (Kruskal–Wallis test, *P*=0.116). (**d**) bAP-induced responses. Upper, representative somatic voltage trace of three APs evoked at 50 Hz (single recording). Middle, spine Ca^2+^ signals before (black) and after application of AP5 (blue). Lower, spine Ca^2+^ signals before (black) and after application of Ni^2+^ and nimodipine (green). (**e**) Summary of the effect of AP5 (blue) and VGCC blockers (green) on bAP-evoked spine (AP5: *n*=35, *P*=0.461; VGCC blockers: *n*=24, *P*<0.001; 3–4 spines per dendrite, Wilcoxon test) and shaft (AP5, *n*=10 dendrites, *P*=0.507; VGCC blockers: *n*=7 dendrites, *P*<0.05, Wilcoxon test) Ca^2+^ signals. Filled symbols and error bars represent mean±s.e.m. Ctr, control; diff., difference in; exp., expected; nonlin., nonlinearity; nimo, nimodipine.

Blockade of NMDARs by 50–100 μM AP5 strongly reduced 4S spine Ca^2+^ signals and eliminated spine Ca^2+^ nonlinearity at distal locations (Fig. 3a,b; Supplementary Fig. 1g), while decreasing EPSP summation modestly (Fig. 3c). In contrast, a combination of T-, R- and L-type VGCC inhibitors (100 μM Ni^2+^ and 10 μM nimodipine^17^) had no significant effect on Ca^2+^ nonlinearity (Fig. 3a,b). Dendritic Ca^2+^ signals were also eliminated by AP5, but not significantly affected by the VGCC blockers (control, mean±s.e.m.: 71±12%, median: 52%; AP5, mean±s.e.m.: 16±2%, median: 16%, *P*<0.001; VGCC blockers: mean±s.e.m.: 48±5%, median: 50% Δ*F*/*F*; *P*=0.198, multiple comparisons after Kruskal–Wallis test with *P*<0.001). As a control, VGCC inhibitors (but not AP5) significantly reduced backpropagating AP (bAP)-evoked spine and shaft Ca^2+^ signals^15^,^17^,^19^ (Fig. 3d,e). We found no involvement of Ca^2+^ release from intracellular stores in 4S spine Ca^2+^ nonlinearity (Supplementary Fig. 3g). These results demonstrate that NMDARs are responsible for cooperativity of spine Ca^2+^ signalling. Although we blocked VGNCs in these experiments, similar cooperative spine Ca^2+^ nonlinearity was measured at distal dendritic locations without TTX as well (Supplementary Fig. 4c,d), with location-independent EPSP summation (Supplementary Fig. 4c,d), indicating that the 4S condition was subthreshold to dendritic Na^+^ spike generation even at distal sites, as expected^22^. Clustered multisynaptic activity can trigger regenerative NMDAR-mediated spikes in thin dendrites, characterized by a slow supralinear voltage component^20^,^22^,^25^,^26^,^27^,^28^,^30^. However, the four physiological-sized inputs used here apparently did not evoke such regenerative events even at the tip, because we found neither larger peak EPSP nonlinearity (Fig. 1c,f,h, Fig. 2b) nor substantial prolongation of EPSPs (half width_measured_/half width_calculated_, proximal: 1.08±0.03, *n*=16; distal: 1.17±0.03, *n*=34, *P*=0.082, Mann–Whitney test) in distal than in proximal compartments. The experimentally observed proximodistal gradients of spine Ca^2+^ and EPSP nonlinearities were replicated in a morphologically detailed CA1PC model with passive dendrites (Supplementary Fig. 5). In summary, NMDAR-mediated spine Ca^2+^ signals are highly sensitive to coincident activation of even low numbers of spatially close synapses in distal dendritic compartments. This local cooperative function takes place in the linear electrical integration regime, where voltage recordings at the soma remain uninformative about the spatial distribution or cooperation of the synapses involved.

### Spatiotemporal requirements for cooperativity

**Figure 4:**
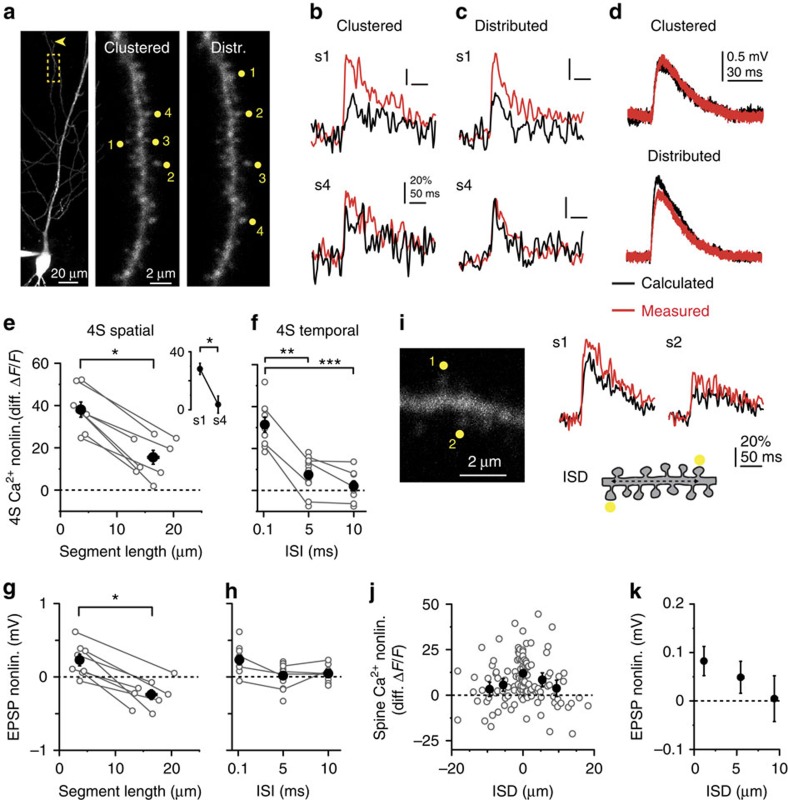
Spatiotemporal properties of cooperative spine Ca^2+^ signalling in distal compartments. (**a**) Left: low-magnification *z*-stack of a CA1PC, stimulation site on oblique dendrite indicated by yellow box. Arrowhead points to dendrite tip. Middle and right: high-magnification *z*-stack of the stimulated segment, with clustered (middle) and distributed (right) arrangement of inputs. Distributed inputs were always activated distal to proximal. (**b**,**c**) Representative Ca^2+^ signals from spine #1 (upper) and spine #4 (lower) in clustered (**b**) and distributed (**c**) arrangement using the 4S protocol (ISI=0.1 ms). (**d**) Integrated somatic EPSPs corresponding to (**b**,**c**). In **b**–**d**, black traces represent calculated responses and red traces represent measured responses. (**e**) Spine Ca^2+^ nonlinearity (average of all four spines) in clustered versus distributed arrangement (*P*<0.05, Wilcoxon test). Grey lines represent experiments in individual dendrites. Inset: Ca^2+^ nonlinearity from spine #1 (first, most distal) and #4 (last, most proximal) in experiments with distributed input. (**f**) Dependence of Ca^2+^ nonlinearity on input synchrony using clustered arrangement in distal dendritic segments (*n*=9–14 spine sets in 17 dendrites, seven cells, multiple comparisons after Kruskal–Wallis test with *P*<0.001). Grey lines represent experiments on the same spine set. (**g**,**h**) Somatic EPSP nonlinearity as a function of the dendritic segment length for spatial distribution (**g**, *P*<0.05, Wilcoxon test), and of ISI (**h**, clustered stimulation, ISD<6 μm, no significant differences with multiple comparisons after Kruskal–Wallis test with *P*=0.044). (**i**) Left: single 2P image of a distal segment with two stimulated spines indicated. Right: Ca^2+^ signals in the two spines during synchronous stimulation. Cartoon depicts measurement of interspine distance (ISD). (**j**) Ca^2+^ cooperativity among spine pairs as a function of ISD (*n*=122 spines). Positive or negative ISD value represents spine sequence towards tip or soma, respectively. Symbols represent mean±s.e.m. for data binned in 0±2.5, 2.5–7.5 and 7.5–12.5 μm. Kruskal–Wallis test for 0±2.5 (*n*=62), 2.5–7.5 (*n*=27) and 7.5–12.5 μm (*n*=23) bins, *P*=0.003. (**k**) EPSP nonlinearity as a function of ISD (grouped by absolute ISD values binned as in **j**). Filled symbols and error bars represent mean±s.e.m. distr., distributed; nonlin., nonlinearity.

We next explored the spatial and temporal requirements for subthreshold cooperation of NMDAR-mediated spine Ca^2+^ signals in distal dendritic compartments. When four synchronously activated inputs were evenly spread on an approximately 15–20-μm-long dendritic segment close to the tip, average spine Ca^2+^ cooperativity decreased but still remained substantial (Fig. 4a–c,e), with decreasing distal-to-proximal nonlinearity profile in individual spines (Fig. 4e)^26^,^27^. Next, four clustered inputs were activated with variable synchrony. Inputs with 5–10 ms ISI produced smaller Ca^2+^ nonlinearity than those with 0.1 ms ISI (Fig. 4f), even though dendritic Ca^2+^ signals did not decrease (ISI 0.1 ms: 46±9%, *n*=9; 5 ms: 39±4%, *n*=14; 10 ms: 36±3%, *n*=9; Kruskal–Wallis test: *P*=0.931). This coincidence requirement suggests that the slower mechanisms such as diffusion are unlikely to contribute substantially to the Ca^2+^ nonlinearity. EPSP summation by four inputs showed similar albeit weaker dependence on spatiotemporal input arrangement (Fig. 4g,h). Finally, stimulating only two synapses we found small but detectable Ca^2+^ cooperativity between synchronously activated spines located within ∼5–10 μm (Fig. 4i–k).

### Relation to A-type K^+^ channels and dendritic spike strength

**Figure 5:**
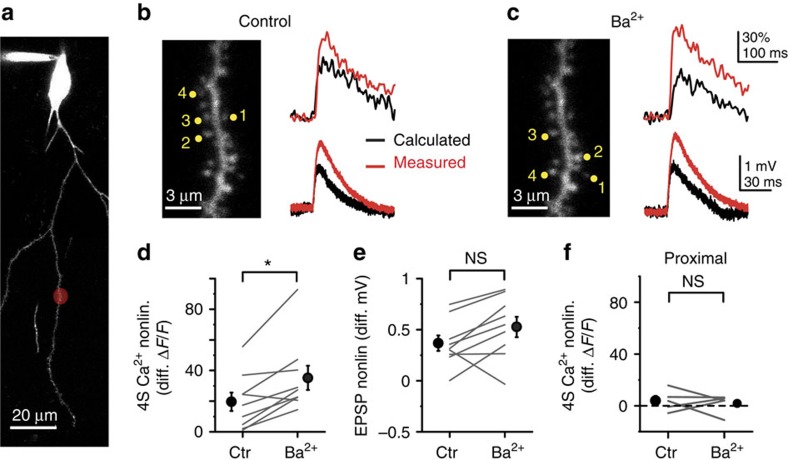
Ba^2+^-sensitive K^+^ channels regulate cooperative synaptic Ca^2+^ signalling. (**a**) Partial 2P *z*-stack of a basal dendrite in a CA1PC, with the stimulation site (∼30 μm distal from branch point) indicated by red spot. (**b**) 4S protocol on a set of four spines under control conditions at the stimulation site shown in (**a**). Left: stimulated spines. Right: spine Ca^2+^ signals (upper, averaged data from the four spines) and somatic EPSP (lower). Black traces: calculated responses; red traces: measured responses. (**c**) 4S protocol on another nearby spine set in the same segment (s3 is the same as s2 in **b**) after bath application of 250 μM Ba^2+^. (**d**) Summary of the effect of 200–250 μM Ba^2+^ on spine Ca^2+^ nonlinearity (*n*=9, *P*<0.05, Wilcoxon test). Grey lines represent individual dendrites, where 1–2 nearby spine sets (data averaged) were measured under control versus Ba^2+^-treated conditions. Stimulation sites were 29±3 μm distal from the branch point, mostly at the middle of dendrites (0.53±0.05 relative distance along branch). (**e**) Somatic EPSP nonlinearity in the corresponding experiments (*n*=9, *P*=0.066, Wilcoxon test). (**f**) No effect of 200 μM Ba^2+^ on spine Ca^2+^ nonlinearity in proximal compartments (0.18±0.03 relative distance along branch, *n*=5, *P*=0.500, Wilcoxon test). Filled symbols and error bars represent mean±s.e.m. Ctr, control; nonlin., nonlinearity.

**Figure 6:**
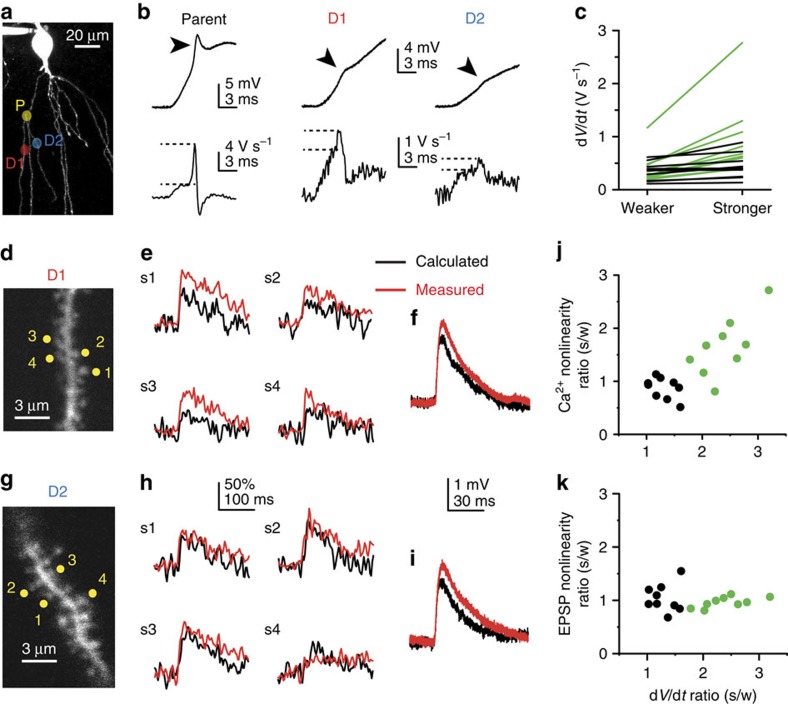
Spine Ca^2+^ cooperativity correlates with dendritic Na^+^ spike strength. (**a**) Partial 2P *z*-stack of a CA1PC, with stimulation sites on terminal sister dendrites (D1 and D2) branching off from a common parent dendrite (P) indicated. (**b**) Representative voltage traces (upper) and corresponding d*V*/d*t* (lower) of dendritic Na^+^ spikes (pointed at by arrowheads) evoked at the stimulation sites in the three dendrites indicated in **a** (see Methods). Note that parent branch spike is much stronger than daughter branch spikes. All dendritic families used in the analysis had parent branch spike with d*V*/d*t*>2 V s^−1^. (**c**) Comparison of dendritic Na^+^ spike strength between terminal sister branches. Black: d*V*/dt_stronger branch_/d*V*/dt_weaker branch_<1.7 (*n*=9); green: d*V*/d*t*_stronger branch_/d*V*/d*t*_weaker branch_>1.7 (*n*=9). (**d**) Stimulated spine set in D1 shown in **a** and **b**. (**e**) 4S protocol on the four spines in D1 shown in **d**. (**f**) Somatic EPSP nonlinearity corresponding to the experiment in **e**. (**g**–**i**) same as **d**–**f** but in D2 shown in **a** and **b**. (**j**,**k**) Ratio of spine Ca^2+^ nonlinearity (**j**, Spearman *R*=0.671, *P*<0.05, *n*=18) and somatic EPSP nonlinearity (**k**, Spearman *R*=−0.003, *P*=0.990, *n*=18) by the 4S protocol in connected sister branches, as a function of the ratio of their d*V*/d*t* (Na^+^ spike strength). Black and green dots indicate homogenous and heterogeneous pairs, respectively.

Dendritic excitability and integration of multiple synaptic inputs is controlled in a compartmentalized fashion by transient A-type K^+^ currents (*I*_A_) in CA1PCs^22^,^23^,^24^,^49^. We next asked how *I*_A_ affects spine Ca^2+^ cooperativity. Partial inhibition of *I*_A_ by 200–250 μM Ba^2+^ (ref. 50) increased nonlinearity of spine Ca^2+^ signals by the 4S protocol (Fig. 5a–d) with a tendency to similarly affect EPSP nonlinearity (Fig. 5e) at middle-distal, but not at proximal (Fig. 5f) dendritic locations. Previous studies revealed that *I*_A_ activity, and thereby dendritic Na^+^ spike propagation strength can vary even between sister dendrites branching from the same parent dendrite, indicating branch-specific *I*_A_ regulation^23^,^24^. We thus compared cooperative nonlinearity of spine Ca^2+^ signals in pairs of terminal sister branches originating from strong spiking (somatic d*V*/d*t*>2 V s^−1^) parent dendrites (Fig. 6; see Methods). We chose to compare sister branches to ensure as similar morphological and passive dendritic properties, and electrotonic distance from the soma as possible. The ratio of Na^+^ spike d*V*/d*t* in sister dendrites was variable (Fig. 6a–c) consistent with independent regulation of spike strength in individual branches^23^. We operationally separated sister branch pairs to those with >70% difference in Na^+^ spike strength (heterogeneous pairs, d*V*/d*t* in stronger branch: 0.964±0.247 V s^−1^, weaker branch: 0.401±0.103 V s^−1^, *n*=9; stronger/weaker d*V*/d*t* ratio 2.40±0.14) and those where spike strength was comparable (similar pairs, <70% difference; d*V*/d*t* in stronger: 0.442±0.081 V s^−1^, weaker: 0.343±0.059 V s^−1^, *n*=9; stronger/weaker d*V*/d*t* ratio 1.30±0.07; Fig. 6a–c)^23^. Experimentally adjustable parameters were similar between heterogeneous sister branches (see Methods). d*V*/d*t* ratio of sister branches did not depend on their length ratio (Spearman *R*=0.411, *P*=0.089, *n*=18) and no obvious morphological differences between sister branches were detected (although diameter or spine density cannot be precisely measured using 2PI), suggesting that spike strength difference could be caused by different *I*_A_ activity^23^,^24^. After measuring Na^+^ spike strength, we measured cooperative spine Ca^2+^ nonlinearity using the 4S protocol in both sister dendrites (in 1 μM TTX, data averaged from 1 to 3 sets of four synchronously activated spines per branch; Fig. 6d–i). We found a correlation between the Na^+^ spike d*V*/d*t* ratio and the ratio of cooperative spine Ca^2+^ nonlinearity, with larger spine Ca^2+^ nonlinearity in stronger than in weaker spiking sister branches in the heterogeneous group (Fig. 6j; stronger: 19.84±3.04%, weaker: 13.87±2.93%, *n*=9, *P*=0.038, Wilcoxon test) but not among similar sister pairs (stronger: 21.49±2.65%, weaker: 26.28±4.29%, *n*=9, *P*=0.109, Wilcoxon test). In contrast, the EPSP nonlinearity ratio did not correlate with the d*V*/d*t* ratio of sister pairs (Fig. 6k). Thus, compartmentalized differences in *I*_A_ activity can translate into variable strength of correlated subthreshold (spine Ca^2+^) and suprathreshold (dendritic Na^+^ spike)-mediated synaptic coincidence detection in individual branches.

### Location-dependent cooperative synaptic LTP

**Figure 7:**
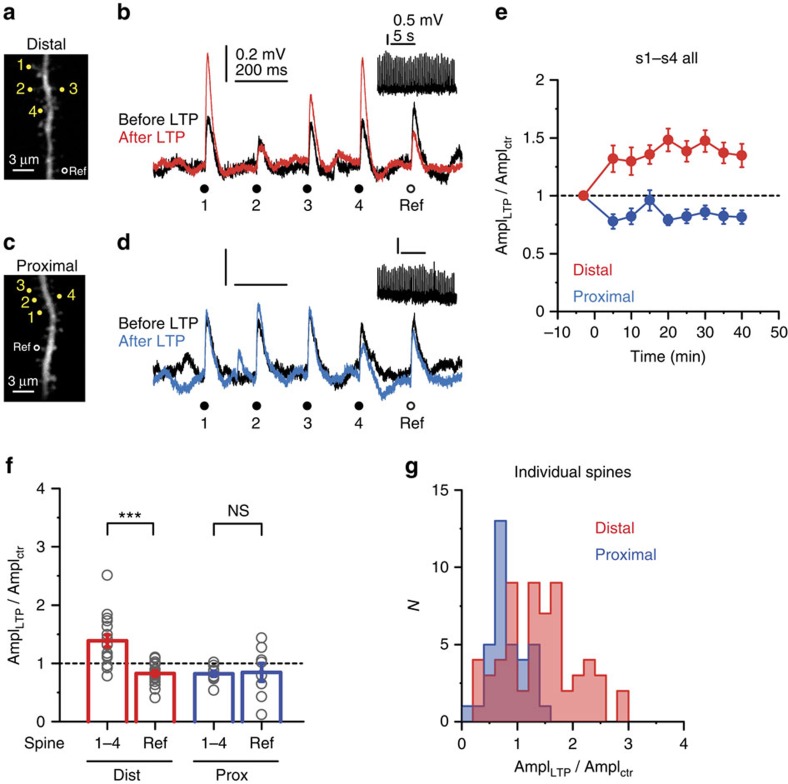
Cooperative synaptic LTP in distal but not in proximal dendritic segments. (**a**) 2P image of a distal segment in an oblique branch. Spines s1–s4 were included in the LTP protocol; the reference spine did not receive LTP induction stimulus. (**b**) Somatic EPSPs by the five spines (s1–s4 (yellow circles) and reference spine (black circle)) in **a**, before (black) and >30 min after (red) delivering the LTP induction protocol to s1–s4 (inset shows LTP induction voltage trace). (**c**,**d**) Similar experiment as in **a** and **b** on a proximal segment in an oblique branch. (**e**) Time course of the effect of cooperative LTP protocol on somatic EPSP amplitude at s1–s4 in distal and proximal dendritic segments. (**f**) Summarized effect of the cooperative LTP protocol on peak somatic EPSP amplitude evoked in distal versus proximal segments in s1–s4 (distal (red), *n*=17 cells; proximal (blue), *n*=10 cells, grey circles represent averaged data from s1–s4 in individual experiments.) and in reference spines (distal (red), *n*=16; proximal (blue), *n*=8, grey circles represent individual spines). (**g**) Histogram of EPSP amplitude change in distal (red) and proximal (blue) s1–s4 spines. Note the normal distribution of the LTP effect in distal spines (Shapiro–Wilks test, *P*=0.120). Group data are presented as mean±s.e.m. ctr, control; Dist, distal; Prox, proximal; Ref, reference.

Spine Ca^2+^ signalling is considered to be fundamental in determining the sign and strength of long-term synaptic plasticity^11^. Cooperative enhancement of spine Ca^2+^ signals in coactive synapses shown above may promote clustered forms of synaptic plasticity, with lowest input threshold in distal high-impedance dendritic compartments. To examine this hypothesis, we measured peak amplitude changes of EPSPs (initial amplitude; proximal: 0.42±0.03 mV, *n*=35 spines; distal: 0.31±0.02 mV, *n*=61 spines) in response to a cooperative 2PGU LTP induction protocol that involved synchronous stimulation of four spatially clustered spines (0.1 ms ISI, 0.5 ms uncaging duration per spine; Supplementary Fig. 6), repeated 50 × at 3 Hz in normal ACSF near the resting membrane potential (∼−64 mV). In most experiments, EPSPs were also measured at an additional nearby (<15 μm) reference spine that was not stimulated during the cooperative LTP protocol. Cells were first loaded with Alexa Fluor 488 via brief (30–60 s) whole-cell recordings, and patched again after allowing 30–100-min recovery period when spines could be clearly visualized throughout the dendritic arbour. This allowed us to induce LTP at identified spines within 5–10 min after membrane rupture, avoiding disruption of the intracellular milieu critical for LTP^51^ (see Methods). The cooperative LTP protocol led to an increase of somatic EPSP amplitude to 139±10% of control values at the four LTP-induced spines (s1–s4) at distal dendritic locations (Fig. 7a,b,e,f, *P*<0.01, one-sample Wilcoxon test, *n*=17 experiments; data from s1 to s4 averaged). The effect was heterogeneous among spines even within spine sets (Fig. 7g; Supplementary Fig. 7a,b), but followed a normal distribution (Fig. 7g). The heterogeneity in LTP depended neither on spine order in the activation sequence (Supplementary Fig. 7a,b) nor on initial EPSP amplitude (Supplementary Fig. 7c). Importantly, EPSP amplitude did not increase (in fact, slightly decreased) in the reference spine that was not stimulated during the LTP protocol, indicating input specificity of potentiation (Fig. 7a,b,f; 83±5% of control, *n*=16, *P*<0.01, one-sample Wilcoxon test; comparison with LTP spines: *P*<0.001, Wilcoxon test). While these experiments were performed mostly on apical oblique dendrites, in an extended data set we found similar cooperative LTP in basal distal segments (apical: 131±10%, *n*=18; basal: 152±14%, *n*=6, *P*=0.193, Mann–Whitney test). LTP was only evoked when both caged glutamate and uncaging laser pulses were presented during the induction protocol (Supplementary Fig. 7d). In contrast to distal locations, no cooperative LTP could be induced at proximal dendritic locations using the same protocol; instead, a long-lasting slight decrease of EPSP amplitude was observed (Fig. 7c–g; 82±4% of control, *n*=10, *P*<0.01, one-sample Wilcoxon test) that did not differ from the amplitude change at reference spines (85±16% of control, *n*=8, *P*=0.888, Wilcoxon test).

**Figure 8:**
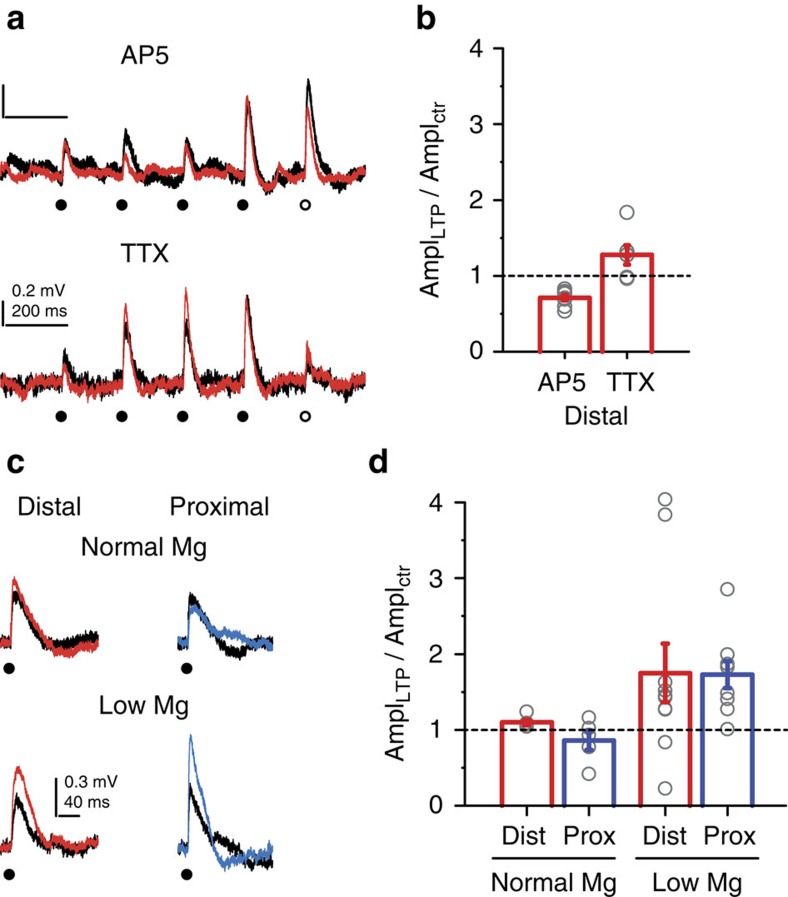
LTP is mediated by voltage-dependent NMDARs but not Na^+^ channels. (**a**) Representative cooperative LTP experiment at a distal oblique segment in the presence of 50 μM AP5 (upper) or in the presence of 1 μM TTX (lower). (**b**) Summary of the effect of AP5 (*n*=8 cells, *P*<0.001 for comparison with control, multiple comparisons after Kruskal–Wallis test with *P*<0.001) and TTX (*n*=6 cells, *P*=0.649 for comparison with control) on cooperative LTP at distal locations. Grey circles: averaged data from s1–s4 in individual experiments. (**c**) Representative EPSPs before (black) and >30 min after a single spine LTP protocol at distally (red) and proximally (blue) located spine in normal ACSF (upper traces) and in ACSF containing 0.1 mM Mg^2+^ (lower traces). (**d**) Summary of single spine LTP experiments in normal ACSF (distal, *n*=5 spines; proximal, *n*=5 spines) and in 0.1 mM Mg^2+^ ACSF (distal, *n*=10 spines, proximal, *n*=9 spines). Grey circles: individual spines. Group data are presented as mean±s.e.m. Two-way ANOVA: no interaction between location and Mg^2+^ treatment, *P*=0.735, *P*=0.681 for location, *P*<0.05 for Mg^2+^ treatment. ctr, control; Dist, distal; Prox, proximal.

Cooperative LTP in distal dendritic segments was eliminated by 50 μM AP5, but was not significantly affected by 1 μM TTX, demonstrating that NMDARs but not VGNCs are required for LTP (Fig. 8a,b). Finally, to examine whether the difference in LTP between proximal and distal locations is due to the difference in voltage-dependent alleviation of Mg^2+^ block of NMDARs, we stimulated single spines alone with an LTP induction protocol with the same activity pattern (50 × at 3 Hz) at distal and proximal locations in low Mg^2+^ (0.1 mM) containing ACSF. Consistent with previous studies^51^,^52^ this single spine protocol indeed induced LTP in most spines at both distal and proximal locations (Fig. 8c,d). In contrast, using the same induction protocol in normal ACSF (containing 1 mM Mg^2+^), spines stimulated alone failed to undergo LTP at both locations (Fig. 8c,d; two-way ANOVA: no interaction between location and Mg^2+^ treatment, *P*=0.735, *P*=0.681 for location, *P*<0.05 for Mg^2+^ treatment). These results together indicate that LTP induction and expression are functional in both proximal and distal spines, and suggest that the larger dendritic depolarization generated by coactive inputs in distal, high-impedance dendritic compartments was sufficient to unblock NMDARs and produce cooperative LTP even with low number of clustered inputs.

## Discussion

Using 2PGU to stimulate individual spines, we investigated the fine-grained interactions among small groups of spatially clustered synapses in thin perisomatic dendrites of CA1PCs. We found that the threshold for spine Ca^2+^ supralinearity by spatiotemporally clustered synapses (1) is lower than that of voltage response supralinearities, (2) depends on dendritic location of the cluster with a decreasing proximodistal gradient and (3) is coregulated with dendritic Na^+^ spike propagation strength. Furthermore, we showed that (4) small clusters of distally located synapses can undergo cooperative LTP without dendritic spike generation.

Our results demonstrate that NMDAR-mediated Ca^2+^ signals in individual spines are highly sensitive to the spatiotemporal activity pattern of even a few nearby synapses. In distal segments of perisomatic dendrites, surprisingly few coactive synapses (two to four) within ∼10–20 μm (<10% of synapses^53^) can efficiently influence each other's function in a cooperative manner. This small cluster size is physiologically relevant according to the reports observing similar clusters of coactive synapses during spontaneous network activity^2^,^3^,^4^ or newly potentiated synapses on sensory experience^7^,^8^,^9^, and is similar to the cluster size proposed to be optimal for NMDAR-rich synapses^54^. The cooperative amplification of spine Ca^2+^ signals is produced by a graded NMDAR-mediated mechanism^25^, most likely due to effective propagation of EPSPs between adjacent spines in distal compartments, alleviating the Mg^2+^ block of NMDARs and leading to supralinear Ca^2+^ influx in coactive nearby spines. Because dendritic depolarization by a synapse depends on dendritic impedance, this mechanism is expected to be location dependent. Indeed, the dendritic map of synaptic Ca^2+^ cooperativity in perisomatic dendrites is consistent with the differences in local biophysical dendritic properties. In high-impedance terminal segments, small spine-to-dendrite voltage attenuation produces strong dendritic depolarization^17^,^18^,^47^, allowing even a few closely located inputs to interact, whereas larger numbers of inputs are necessary to evoke nonlinear amplification of synaptic Ca^2+^ signals at low-impedance proximal dendritic locations. Indeed, the same mechanism affects dendritic spike properties depending on the input location and spatial pattern^26^,^27^,^28^,^29^. Here we show the impact of dendritic location on local cooperativity of spine Ca^2+^ signals, without engaging more global dendritic spikes. In fact, an interesting feature of the spatial gradient in the subthreshold scenario is a dissociation of nonlinearity in electrical versus Ca^2+^ signalling; while nonlinear spine Ca^2+^ signals increased in the proximodistal direction along branches, somatic EPSP integration was largely location independent. This is likely due to the different effect of depolarization on the AMPA receptor (AMPAR) current, mainly producing EPSPs, and on NMDARs, mainly responsible for spine Ca^2+^ signals. Depolarization by an active synapse decreases AMPAR current in neighbours through a reduction in driving force, counterbalanced by increased NMDAR current. In contrast, Ca^2+^ influx through NMDARs increases as a net effect of NMDAR conductance increase by Mg^2+^ unblock and only little change in Ca^2+^-driving force. Thus, local interactions in Ca^2+^ signalling among synapses may remain virtually undetectable in somatic voltage recordings as long as the compound depolarization remains subthreshold to local spikes.

Our results, including the relative location independence of mEPSP amplitude and individual spine Ca^2+^ signals as well as the increasing proximodistal gradient of spine Ca^2+^ cooperativity along thin dendrites, are consistent with relatively uniform synaptic properties in dendritic spines, and dendritic depolarization varying with location^17^,^18^. However, systematic distance-dependent differences of synaptic or voltage-dependent dendritic properties along thin branches may also influence location dependence of Ca^2+^ cooperativity. While such spatial distributions of voltage-gated channels are mostly unexplored, different gradients of synaptic density and strength along perisomatic dendrites of CA1PCs were proposed to counterbalance the inequality of synaptic strength due to impedance differences within a branch^46^,^53^. Nevertheless, unless the impact of the dendritic impedance gradient is completely neutralized (which is not reached even by twofold decrease of synaptic strength from branch base to tip (ref. 53; Supplementary Fig. 5d,e), spine Ca^2+^ cooperativity is expected to increase gradually along these terminal dendrites.

The factors determining cooperativity of Ca^2+^ signalling and plasticity among synchronously active nearby synapses are incompletely understood^4^,^17^,^39^,^40^,^42^. Cooperative Ca^2+^ signalling in coactivated adjacent spines, mediated by NMDARs, was observed in striatal medium spiny neurons^39^ and CA1PCs^17^ using 2PGU, but its location dependence was not studied. In contrast, synaptic Ca^2+^ signal amplitude was not sensitive to the activity of nearby synapses in barrel cortex L4 spiny stellate cells^40^ or developing visual cortex L2/3 PCs^4^ *in vivo* and in CA3PCs in slice cultures^4^. In mouse hippocampal slices, synchronous stimulation of more than ∼12 spines by 2PGU was required to induce clustered LTP (measured by spine volume increase) at relatively proximal CA1PC oblique dendrites^42^, consistent with the lack of LTP by four inputs at similar locations in our experiments. On the basis of the present results, we expect that besides dendritic location, subtle differences in dendritic and synaptic morphology as well as in Ca^2+^ signalling components may influence synaptic Ca^2+^ cooperativity depending on the cell type, synapse type and developmental stage.

Cooperative Ca^2+^ signalling may have many functional consequences, including a role in synaptic plasticity. Induction of canonical LTP at Schaffer collaterals requires sufficient postsynaptic depolarization during synaptic NMDAR activation producing large Ca^2+^ signals in the spine head^11^,^55^. The necessary depolarization can be provided by properly timed bAPs or by robust and coincident synaptic activity, rendering LTP cooperative/associative^11^,^33^,^34^,^35^,^36^,^56^,^57^. However, cooperativity among inputs was investigated mostly at low spatial resolution using electrical stimulation^12^,^33^,^34^,^35^,^58^. Therefore, the number and spatiotemporal pattern of coactive synapses required to trigger cooperative LTP has not yet been determined, and the nature of the underlying dendritic event remained elusive. We show that, in terminal perisomatic dendritic segments, as few as four coactive clustered Schaffer collateral synapses can induce cooperative LTP, which is rapid, input specific (and therefore not due to heterosynaptic plasticity or changes in general dendritic properties), NMDAR dependent, and maintained for ⩾40 min, matching essential features of canonical Schaffer collateral LTP^11^,^55^. This plasticity, if accompanied by structural remodelling^59^ and longer survival of the synapses involved^60^, is well suited to promote activity-dependent clustering of inputs carrying correlated information. Such connectivity patterns may increase information-storing capacity of neurons through exploitation of nonlinear dendritic integration^61^, and accumulating evidence indicates synaptic clustering during development and learning in certain neuron types (reviewed recently^62^,^63^) including CA1PCs^1^. According to our results, terminal segments of thin and long dendrites may be particularly suited for cooperative plasticity of small synaptic clusters. Interestingly, experience-induced enrichment of GluA1-containing AMPARs indeed occurs in spine clusters preferentially at distal parts of dendrites in barrel cortex L2/3 PCs^9^. While we found no cooperative LTP with four clustered synapses at proximal dendritic segments, we expect that cooperative plasticity requires gradually increasing synapse cluster size along the distal-to-proximal dendritic axis. Alternatively, proximal and distal synapses may follow different synaptic plasticity rules^64^, a hypothesis requiring further investigation.

Clustered forms of synaptic plasticity, mediated by protein synthesis-dependent synaptic tagging^42^ or diffusible intracellular molecules^65^, were shown between nearby, sequentially activated strong and weak synaptic inputs^41^,^42^. However, in these plasticity studies, the temporal window for input co-occurrence was on the order of minutes; therefore, these synapses may belong to different information-coding ensembles that are contextually related in a temporal sequence of events. In contrast, cooperative LTP of synchronous clustered synapses, as shown in the present study, is suitable to bind truly coincident inputs, presumably coding the same environmental feature, on the same dendritic segment. Potentiation and/or stabilization of clustered synapses with highly correlated activity may be in fine balance with heterosynaptic depression of nearby uncorrelated or inactive synapses^4^,^43^. Thus, a complex mixture of different pattern-specific learning rules may collectively allow dynamic, activity-dependent rearrangement of synaptic strength in local dendritic subcompartments. Intriguingly, we found considerable heterogeneity in the degree of potentiation among individual spines by the LTP protocol, and EPSP amplitude even decreased in a number of spines. Decrease of amplitude was also observed under several conditions evoking no net LTP (for example, in proximal spines, in distal reference spines or in AP5). Beside possible technical reasons (for example, intracellular dialysis^51^), this may be explained, for example, by variable history of spines, confounding LTD-inducing effect of the low-frequency test stimuli or homeostatic plasticity.

Dendrites of CA1PCs can produce regenerative spikes *in vivo*^5^,^6^, and VGNC/NMDAR-mediated local dendritic spikes can be activated in perisomatic dendrites by robust clustered synaptic input *in vitro*^21^,^22^,^23^. Dendritic spikes are implicated as local coincidence detection signals important for induction of synaptic plasticity^11^,^33^,^34^,^35^,^36^. However, we found no indication for generation or requirement of bAPs or dendritic spikes for cooperative spine Ca^2+^ signalling or plasticity: (1) somatic voltage responses by the 4S protocol showed no substantial transient or prolonged component compared with calculated responses (as hallmarks for dendritic spikes); (2) voltage and Ca^2+^ signals increased monotonically with each added input without an apparent threshold; and (3) cooperative Ca^2+^ signalling and LTP could be induced in distal compartments in the presence of TTX, ruling out a role for Na^+^ spikes generated by our stimulation protocol. Thus, the graded boosting effect of synaptic Ca^2+^ signals by NMDARs^25^ is the most likely mechanism to underlie the observed effects. This form of cooperative plasticity, which dissociates the threshold for inducing LTP from the higher threshold for local spike generation, may link the strength of the synapse to activity of its closest neighbours instead of a more global output of the dendrite or the soma, allowing the storage of initially subthreshold input patterns with potential consequential transition from linear to supralinear integration of learned patterns. Interestingly, recent theoretical work predicted an increase of storage capacity with two dendritic learning thresholds: a lower synaptic learning threshold and a higher dendritic spike threshold^66^.

Our results revealed a relationship between synaptic cooperativity and dendritic excitability. First, inhibition of (presumably A-type) K^+^ currents increased synaptic cooperativity; second, cooperativity correlated with somatic strength of dendritic Na^+^ spikes. *I*_A_, whose function depends on previous local activity^23^,^49^,^67^, was implicated in compartmentalized long-term enhancement of dendritic Na^+^ spike propagation^23^,^24^. This delineates a positive-feedback loop, whereby clustered synaptic activity on a dendritic segment enhances branch excitability, which in turn could facilitate potentiation of coincident nearby synapses through increased synaptic cooperativity as well as through enhanced regenerative spike-based coincidence detection mechanisms^22^,^49^. The reciprocal interdependence of synaptic and dendritic functions may lead to complex rules for processing and storage of information represented by correlated synaptic input patterns.

## Methods

### Hippocampal slice preparation and patch-clamp recordings

Adult male Wistar rats (7–11-week old) were used to prepare transverse slices (400 μm) from the hippocampus similarly to that described previously^22^, according to methods approved by the Animal Care and Use Committee of the Institute of Experimental Medicine, Hungarian Academy of Sciences, in accordance with 86/609/EEC/2 and DIRECTIVE 2010/63/EU Directives of the European Community. Slices were incubated in a submerged holding chamber in ACSF at 36 °C for 30 min, and then stored in the same chamber at room temperature. For recording, slices were transferred to a custom-made submerged recording chamber under the microscope where experiments were performed at 32–35 °C in ACSF containing (in mM): NaCl 125, KCl 3, NaHCO_3_ 25, NaH_2_PO_4_ 1.25, CaCl_2_ 1.3, MgCl_2_ 1, glucose 25, Na-pyruvate 3 and ascorbic acid 1, saturated with 95% O_2_ and 5% CO_2_. In low-Mg^2+^ LTP experiments, MgCl_2_ was decreased to 0.1 mM. Cells were visualized using an Olympus BX-61 or a Zeiss Axio Examiner epifluorescent microscope equipped with differential interference contrast optics under infrared illumination and a water immersion lens (× 60, Olympus or × 63, Zeiss). Current-clamp whole-cell recordings from the somata of hippocampal CA1PCs were performed using a BVC-700 (Dagan, Minneapolis, MN, USA) or an EPC800 (HEKA) amplifier in the active ‘bridge' mode, filtered at 3–5 kHz and digitized at 50 kHz. Patch pipettes (2–6 MΩ) were filled with a solution containing (in mM): K-gluconate 134, KCl 6, HEPES 10, NaCl 4, Mg_2_ATP 4, Tris_2_GTP 0.3, phosphocreatine 14 (pH=7.25), complemented with either the Ca^2+^-sensitive dye Oregon Green BAPTA-1 (OGB-1, 100 μM) and Alexa Fluor 594 (50 μM; all fluorescent dyes from Invitrogen–Molecular Probes) or Alexa Fluor 488 (100 μM, LTP experiments). In some Ca^2+^ measurements (Supplementary Fig. 4a,b), the lower-affinity Ca^2+^ dye Fluo-5F (300 μM; *K*_d_∼550 nM (ref. 19)) and Alexa Fluor 594 (10 μM) was used. Series resistance was <30 MΩ. Voltages were not corrected for liquid junction potential. Only CA1 neurons with a resting membrane potential more negative than −55 mV were used. Cells were kept at −63– −65 mV.

### Two-photon imaging and uncaging

A dual-galvanometer-based two-photon scanning system (Prairie Technologies, Middleton, WI, USA) was used to image the neurons and to uncage glutamate at individual dendritic spines^17^,^22^,^23^,^24^. Two ultrafast pulsed laser beams (Chameleon Ultra II; Coherent, Auburn, CA, USA) were used, one for imaging (at 920 nm for OGB-1 and Alexa Fluor 488 or at 880 nm for imaging Alexa Fluor 594) and the other to photolyse MNI-caged L-glutamate at 720 nm (Tocris; 10 mM, applied through a puffer pipette with an approximately 20–30-μm diameter, downward-tilted aperture above the slice using a pneumatic ejection system (PDES-02TX (NPI, Tamm, Germany). Laser beam intensity was independently controlled with electro-optical modulators (Model 350–50, Conoptics, Danbury, CT, USA). Emitted light was collected by multi-alkali or GaAsP photomultipliers (Hamamatsu Photonics K.K, Iwata City, Japan).

All neurons included in the study had largely complete apical and basal dendritic arbours. The selected basal (stratum oriens) and apical oblique (stratum radiatum) dendrites were carefully examined and only complete, uncut branches with >70-μm length were used. We did not detect differences between basal and apical oblique dendrites (Supplementary Fig. 2k) in 4S Ca^2+^ nonlinearity (at middle locations: apical, 22.27±3.28% Δ*F*/*F*, *n*=13; basal, 19.70±1.96% Δ*F*/*F*, *n*=20, *P*=0.645; at distal locations: apical, 33.11±2.94% Δ*F*/*F*, *n*=30; basal, 35.80±6.40% Δ*F*/*F*, *n*=7, *P*=0.712, Mann–Whitney test) or EPSP nonlinearity (at middle locations: apical, 0.56±0.13 mV, *n*=13; basal, 0.48±0.06 mV, *n*=20, *P*=0.617; at distal locations: apical, 0.32±0.07 mV, *n*=30; basal, 0.25±0.20 mV, *n*=7, *P*=0.194, Mann–Whitney test), therefore results from apical and basal branches were pooled. Proximal locations were not tested in basal dendrites due to the usually low spine density in their initial stem segments. Individual spines with an average phenotype and separated from their neighbours were selected for stimulation. Stimulation was performed by uncaging glutamate ≤0.5 μm lateral to the head of visually identified spines, using 0.2 or 0.5 ms uncaging duration. Time interval between spines (termed interspine stimulus interval, ISI) was either 200–305 ms (in control test traces assessing individual voltage and Ca^2+^ responses) or 0.1 ms (synchronous stimulation), unless otherwise indicated (Fig. 4f,h). Unitary EPSPs and spine Ca^2+^ signals were measured repeatedly (usually two to five times) interleaved with synchronous stimulations.

In experiments examining the relationship between dendritic excitability and cooperative synaptic Ca^2+^ signalling (Fig. 6), dendritic Na^+^ spikes were evoked in regular ACSF by synchronously stimulating 5–10 spines on a short (∼10 μm) segment of basal or apical terminal sister dendrites that branched off from the same parent dendrite. Experimentally adjustable parameters such as the distance of input site from branch point (stronger: 32±2 μm, weaker: 35±2 μm, *n*=9, *P*=0.075, Wilcoxon test), calculated EPSP (stronger: 1.41±0.12 mV, weaker: 1.45±0.13 mV, *n*=9, *P*=0.593, Wilcoxon test) and input clustering (dendrite stretch, stronger: 3.5±0.3 μm, weaker: 2.8±0.4 μm, *n*=9, *P*=0.441, Wilcoxon test) were similar between sister pairs with heterogeneous Na^+^ spike strength. Na^+^ spike strength was characterized by the amplitude of the corresponding component on the d*V*/d*t* trace (first derivative of binomially smoothed voltage traces). Sister branch pairs were considered as expressing heterogeneous Na^+^ spike strength if the d*V*/d*t* ratio of the stronger versus weaker branch exceeded 1.7, based on previous results^23^. After measuring Na^+^ spike strength, TTX was washed in and 1–3 sets of four neighbour spines were used to measure cooperative Ca^2+^ nonlinearity in the first spine. To ensure comparable stimulation conditions, cooperativity experiments were included in the analysis only if (1) at least three spines were successfully stimulated; (2) calculated EPSPs were <2.1 mV with <30% difference between that measured in the two sister branches; and (3) there was <20% difference in the relative locations of stimulus sites along the sister branches.

### Ca^2+^ measurements

In experiments measuring spine Ca^2+^ signal nonlinearity, the bath solution contained 0.5–1 μM TTX to eliminate nonlinearities arising from dendritic Na^+^ spike activation, except where indicated (Supplementary Fig. 4c,d). Freehand linescan imaging through spines was performed at 200–500 Hz with 8-μs dwell time. At the beginning of the experiment, the set of two to four spines were first stimulated individually (200–305-ms intervals) and the laser power was adjusted to yield physiological unitary EPSPs (Supplementary Fig. 1) and reliable associated spine Ca^2+^ signals. Next, stimulation of various numbers of the selected spines was performed with the same laser power synchronously (0.1 ms ISI for galvo movement, plus 0.2 ms uncaging duration per spine). In some experiments, longer ISIs (5–10 ms; Fig. 4f,h) or uncaging duration (0.5 ms, with 0.1 ms ISI; Figs 7 and 8; Supplementary Fig. 6) were used. In experiments examining the effect of larger proximal input clusters (up to 12 spines, Fig. 2e), Ca^2+^ signals were measured only in the first four spines. Following synchronous stimulation, spines were stimulated individually again to confirm the stability of single spine responses. Recordings were repeated three to five times for each condition. To ensure individual stimulation of spines (Supplementary Fig. 3a–c), uncaging points were placed more than ∼1.2 μm apart (note that spines on opposing sides of the dendrite could be stimulated individually).

Ca^2+^ signals measured with OGB-1 were expressed as Δ*F*/*F*=(*F*−*F*_rest_)/*F*_rest_ × 100. Ca^2+^ signals measured with Fluo-5F were normalized to the Alexa Fluor 594 fluorescence, quantified as Δ*G*/*F*=(*F*_green_−*F*_rest,green_)/(*F*_red_−*I*_dark,red_). To directly compare results with the two dyes (Supplementary Fig. 4b), we used the ratio of the measured and the calculated Ca^2+^ signals.

During pharmacological experiments, drugs were applied in the bath for >10 min. In experiments using Ba^2+^ inhibition of A-type K^+^ currents (Fig. 5), stimulus locations were selected at ∼30 μm from the last branch point (similar to Na^+^ spike tests) and neighbouring spine sets were stimulated under control conditions and after >10 min application of Ba^2+^ in the bath. Note that in these experiments the MNI glutamate puffer solution did not contain Ba^2+^ and therefore the effective local Ba^2+^ concentration during puffing is expected to be lower than that in the bath.

When testing the effects of AP5, Ca^2+^ channel blockers or cyclopiazonic acid (CPA) on spine Ca^2+^ signalling (Fig. 3; Supplementary Fig. 3g), the drugs were included in the puffed MNI glutamate solution as well to ensure maximal efficiency, and separate cells were measured under control conditions (no drug in puffer pipette) and in the presence of the drugs (with drug-containing puffer pipette) from different slices of the same animals. Ca^2+^ signals and nonlinearity in control experiments with or without including 0.01% dimethylsulphoxide (DMSO; solvent of nimodipine) did not differ, therefore data of these two control groups were pooled. The effect of CPA (30 μM) was tested in the same way in a separate set of experiments, where CPA-treated cells (>20 min) were compared with control cells (with the solvent DMSO (0.03%) in different slices from the same animals. To ensure comparable stimulation conditions, only experiments with 1–2.1 mV expected EPSP were included in the analysis of these experiments. VGCC-mediated Ca^2+^ signals were evoked by bAPs (triggered by 3-ms-long suprathreshold somatic current injections, 3 pulses at 50 Hz).

### LTP experiments

To measure changes in synaptic function induced by LTP protocols, we recorded EPSPs evoked by 2PGU in whole-cell current-clamp mode. Although intracellular recordings inevitably disturb the internal milieu of the cell, we chose this configuration instead of measuring solely spine volume for several reasons. First, it was important to ensure that the applied uncaging stimuli produced EPSPs in the physiological range regardless of the depth of the stimulated spines, requiring fine adjustments in uncaging laser power in each experiment. Second, in our experience, the ability to monitor the lack of even subtle signs of photodamage in electrophysiological recordings (see below) provides a necessary confirmation to distinguish plasticity related effects from phototoxicity in each experiment.

To prevent washout of intracellular components by whole-cell dialysis^51^, we developed a method where LTP protocol could be started within 5–8 min after establishing the whole-cell configuration. Neurons (usually three to four per slice) were first patched with a pipette solution containing Alexa Fluor 488 (this dye does not bind Ca^2+^, preserving native intracellular Ca^2+^ signalling). After break-in, the cell interior was dialysed for 30–60 s (usually facilitated by gently blowing into the pipette), and then the pipette was carefully withdrawn. After recovery for 30–100 min, the same cell was patched again guided by fluorescent identification using either 2PI or a camera (Andor Zyla). Success rate for repatching exceeded 90%, and repatched neurons had normal *V*_m_ similar to that measured during the loading (*V*_m_ at loading: −60.3±0.3 mV; *V*_m_ at repatching: −60.5±0.2 mV, *n*=103 cells, *P*=0.348, Wilcoxon test). After establishing cell-attached configuration for the repatch, a proximal (relative distance along branch: <0.4) or distal (relative location along branch: >0.6) fluorescent dendritic segment with one to five clearly isolated nearby dendritic spines was selected and stacked (0.5 μm *z*-steps). Then, the seal was ruptured, *V*_m_ was measured and uncaging started immediately at the selected spines. Uncaging duration was 0.5 ms in most of the LTP experiments. We confirmed that this stimulation method produced EPSPs with similar kinetics and spine Ca^2+^ signals with indistinguishable amplitude and kinetics as uncaging with 0.2-ms duration with higher powers (Supplementary Fig. 6). For LTP experiments, the spines were first stimulated separately with control test pulses (200–305 ms between spines) and the uncaging laser power was adjusted to yield physiological-sized EPSPs on each stimulated spine. After the test recording, the LTP induction protocol (50 stimulations at 3 Hz at a single or a set of four spines, as indicated in the text) was delivered using the same laser power. The LTP induction protocol was followed by control test stimulations of the spines every 5 min using the same laser power. In some experiments, we also included a fifth reference spine that did not participate in the LTP protocol but was stimulated individually during the test pulses before and after LTP. The uncaging locations were chosen manually in the immediate vicinity (<0.5 μm) laterally from the tip of the spine head. The uncaging locations were manually readjusted if necessary between test pulses (every 5 min) due to changes in shape, position of loading-related fluorescence of the stimulated spines. Care was taken not to move the uncaging location closer to the spine head during the experiment, to avoid artificial increases in EPSP amplitudes. Control experiments (Supplementary Fig. 7d) and spines (Fig. 7b,d,f) confirmed no EPSP amplitude increase induced in spines without proper LTP stimulation. In fact, EPSP amplitude showed a slight decrease in control experiments both at proximal and distal locations, consistent with previous reports^51^.

The LTP protocol typically evoked similar EPSPs on all 50 pulses with no sign of regenerative spikes (see examples in Fig. 7b,d). Experiments showing electrophysiological signs of photodamage (sudden large irregular depolarization with uneven and slow repolarization during LTP protocol with consecutive loss of reliable single spine responses, often accompanied by morphological changes including spine contour changes or dendritic swelling) were terminated and excluded from the analysis.

LTP experiments were carried out with no TTX in the bath, unless otherwise indicated. In low-Mg^2+^ experiments, wash-in of ACSF with reduced Mg^2+^ concentration (0.1 mM) was started immediately before seal rupture to establish the whole-cell configuration (∼5–7 min before delivering the LTP induction protocol; control test pulse was measured during this wash-in period), and washout was started immediately after completing the LTP induction protocol. When testing the effect of TTX or AP5, the blocker was applied continuously throughout the experiment beginning 10–20 min before repatching the cell, and the glutamate puffer pipette also contained the inhibitor.

The magnitude of plasticity was quantified as the average normalized change in EPSP amplitude between 30 and 40 min after the LTP protocol. We did not analyse spines with <0.1 mV average initial EPSP amplitude to avoid overestimation of LTP due to division by small numbers. We chose not to measure fluorescence-based spine volume to monitor LTP because Alexa Fluor 488 fluorescence increased in repatched cells during the course of the LTP experiment, due to dialysis from the patch pipette. Occasionally (<5%), we observed a retraction or disappearance of the stimulated spine, usually accompanied by a strong reduction (<40% of the control value) or unreliability of response amplitudes. This seemed to occur independently of the location of the spines or the experimental protocol; therefore, we omitted such spines from the analysis. Spines were excluded also if their head moved close to other neighbouring spines due to the shape or size changes throughout the course of the experiment.

### Chemicals

D-AP5, TTX (all from Tocris), Ba^2+^ and Ni^2+^ (Sigma) were dissolved in distilled water in stock solutions, aliquots were stored at −20 °C (D-AP5 and TTX) or room temperature (Ba^2+^ and Ni^2+^) and dissolved into ACSF on the day of experiment. Nimodipine (Tocris) and CPA (Sigma) stock solutions were prepared in DMSO, aliquots stored at −20 °C and diluted into ACSF before the experiment (DMSO v/v 0.01% and 0.03%, respectively). Solutions containing nimodipine were protected from light.

### Data analysis

Analysis was performed using custom-written macros in IgorPro (WaveMetrics, Lake Oswego, OR, USA). Ca^2+^ and voltage signals were analysed offline using averaged traces of three to eight trials with no smoothing or background subtraction. Some experiments exhibited a transient light or electrical artefact from the uncaging laser or the galvo movement at the start of the stimulation, before voltage and Ca^2+^ signals began to rise, these were excised. Calculated EPSP and Ca^2+^ signal amplitudes were measured offline as the peak of the arithmetic sum of the individual responses (shifted and added, mimicking the same input timing as used experimentally). This method takes into consideration any slight crosstalk of Ca^2+^ signals occasionally occurring between individually stimulated spines.

Ca^2+^ signal amplitude was measured as the maximum average of 5 consecutive points within 50 ms after uncaging. This measurement may slightly overestimate the amplitude (5.4±0.4% Δ*F*/*F* on measured Ca^2+^ traces, 6.4±0.7% Δ*F*/*F* on calculated Ca^2+^ traces, based on same measurement on baseline periods on *n*=38 traces in 10 randomly chosen experiments), but since it is an additive error, it does not affect nonlinearity, which is the difference of measured versus calculated amplitude (difference in nonlinearity with or without correction to baseline: −1.0±4.7% Δ*F*/*F*, *P*=0.19, *n*=38). Therefore, we corrected the amplitude for baseline only where absolute values of Ca^2+^ signal are given but not for measurements of nonlinearity. We considered Ca^2+^ signals detectable if exceeding 10% Δ*F*/*F* (average baseline+2 s.d.). Only spines with detectable Ca^2+^ signals to individual stimuli were included in the analysis of Ca^2+^ cooperativity. In Ca^2+^ cooperativity measurements involving increasing numbers of inputs (Fig. 1), we combined data from spine #1 and #2 (even though they were stimulated in reverse order), because their Ca^2+^ nonlinearity was statistically indistinguishable (Supplementary Fig. 2f in a larger data set). Ca^2+^ traces displayed in the figures were smoothed binomially (*N*=1).

Detailed morphological and distance measurements were performed on dye-loaded neurons using ImageJ (NIH, Bethesda, MD, USA). Distances of input site from the soma or trunk were measured from the approximate midpoint of the input site on stacked images. Interspine distances were measured between spine insertion points to the shaft (either visible or the perpendicular projection of the spine head centre to the shaft) on stacks or single-focal images. Relative distances were measured as the distance of the input site divided by the total branch length, measured from the soma (basal dendrites) or the originating branch point from the trunk (apical oblique dendrites). In cases when the dendrite bifurcated distal to the input site (for example, proximal stimulation sites), we considered the longer daughter for total branch length. Proximal, middle and distal locations were categorized by division of the total dendrite length into thirds for Ca^2+^ measurements.

No statistical methods were used to predetermine sample sizes, but our samples are similar to or exceed those reported in previous publications^17^,^22^,^23^,^24^ and that generally employed in the field. Statistical analysis was performed using Wilcoxon-matched pairs test (two paired groups), one-sample Wilcoxon test (LTP experiments, comparison to median=1), Mann–Whitney *U*-test (two unpaired groups), Kruskal–Wallis test and *post hoc* multiple comparisons with Bonferroni adjustment (multiple unpaired groups), two-way repeated measures ANOVA (Fig. 1g) or two-way ANOVA (Fig. 8d), using Statistica (Statsoft, Tulsa, OK, USA) and OriginPro softwares. All statistical tests were two tailed. In the two ANOVA analyses performed, most data passed the Levine test for homogeneity of variance, except for a minor heterogeneity in one group in each (Levene test *P*=0.027 and 0.023). Correlations were analysed using Spearman rank order correlation. Differences were considered significant when *P*<0.05. In all figures, group data are presented as mean±s.e.m. *P<0.05; **P<0.01; ***P<0.001. No explicit randomization method was used, but experiments comparing different conditions were interleaved (between group) or the order of conditions was varied (within group) wherever possible. The experimenter was usually aware of the experimental condition, except for a subset of control and AP5-treated cooperative LTP experiments at distal dendrites that were performed with the experimenter blind to the treatment, with the same results as non-blind experiments.

### Computational modelling

For details of computational modelling, please see Supplementary Fig. 5.

## Additional information

**How to cite this article:** Weber, J. P. *et al*. Location-dependent synaptic plasticity rules by dendritic spine cooperativity. *Nat. Commun.* 7:11380 doi: 10.1038/ncomms11380 (2016).

## Supplementary Material

Supplementary Information Supplementary Figures 1-7 and Supplementary References
