# Supplementary material for: Location-dependent synaptic plasticity rules by dendritic spine cooperativity
Source: Nat Commun. 2016 Apr 21;7:11380. doi: 10.1038/ncomms11380 (PMC4844677; doi:10.1038/ncomms11380)
Supplement: Supplementary Information — Supplementary Figures 1-7 and Supplementary References [file ncomms11380-s1.pdf]

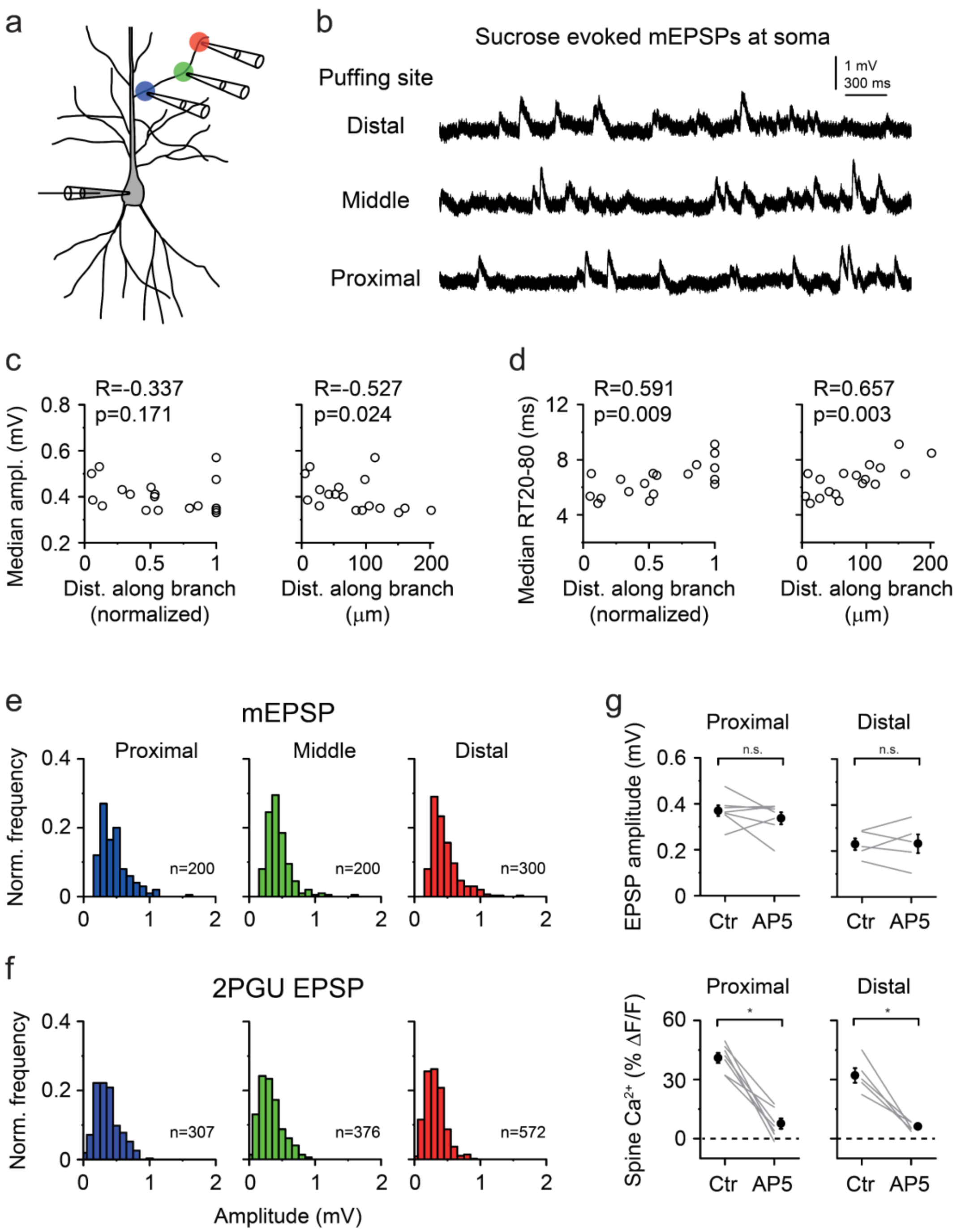

Supplementary Figure 1. Properties of somatic miniature EPSPs and uncaging evoked EPSPs evoked at different dendritic locations.

(**a**) Illustration of sucrose puffing experiments. Miniature EPSPs were evoked by focally puffing a high-osmolarity modified external solution (ACSF with the addition of 300 mM sucrose, 0.5 µM tetrodotoxin and 10 µM gabazine, targeting ~25 µm diameter dendritic area; Magee and Cook, 2000) for 1-3 seconds at various locations along fluorescently identified dendritic segments in Alexa Fluor 488 loaded neurons, while events were recorded at the soma. (**b**) Representative traces of somatically recorded mEPSPs evoked by sucrose puffing at various locations along oblique branches. (**c**) Median amplitude of sucrose-evoked somatic mEPSPs as

a function of normalized (left) and absolute (right) distance of the puffing site along oblique and basal branches. Detection and analysis of mEPSPs was performed using EVAN[1]. Note that somatically measured amplitudes are relatively independent from the puffing site along the branch, consistent with previous simulations[2-3] and similar to synapses located in the apical trunk[4]. (**d**) Median 20-80% rise time of sucrose-evoked somatic mEPSPs as a function of normalized (left) and absolute (right) distance of the puffing site along oblique and basal branches. Note the increase of somatically measured rise time with distance along branches. (**e**) Normalized frequency distribution of mEPSP amplitudes evoked by puffing at proximal, middle and distal dendritic sites. The first fifty events evoked by sucrose puffing (collected from multiple repeated puffs separated by a few minutes) were included from each experiment. Note that small events cannot be reliably detected, resulting in the lack of events with <0.15 mV amplitude. (**f**) Normalized distribution of uncaging evoked EPSPs (2PGU EPSP), stimulated at proximal, middle and distal segments. Note the similarity of 2PGU EPSP amplitude distribution to that of mEPSPs in each compartment. Because the mEPSP amplitude distribution from distal segments may be overestimated compared to proximal segments by losing more slow and small events in the noise, we adjusted our uncaging stimulation of distal synapses to yield slightly smaller EPSP amplitudes than that of proximal synapses (distal inputs: mean ± SEM: 0.31 ± 0.01 mV, median 0.30 mV, n=572; proximal inputs: mean ± SEM: 0.36 ± 0.01 mV, median=0.33 mV, n=307; p<0.005, Kolmogorov-Smirnov test). (**g**) Effect of the NMDAR antagonist AP5 (50 μM) on physiologically sized uncaging evoked somatic EPSPs (upper) and spine Ca^{2+} signals (lower), stimulated at proximal (left) and distal (right) locations. Note that spine Ca^{2+} signal was almost completely eliminated in AP5, whereas the EPSP was not affected (Wilcoxon tests).

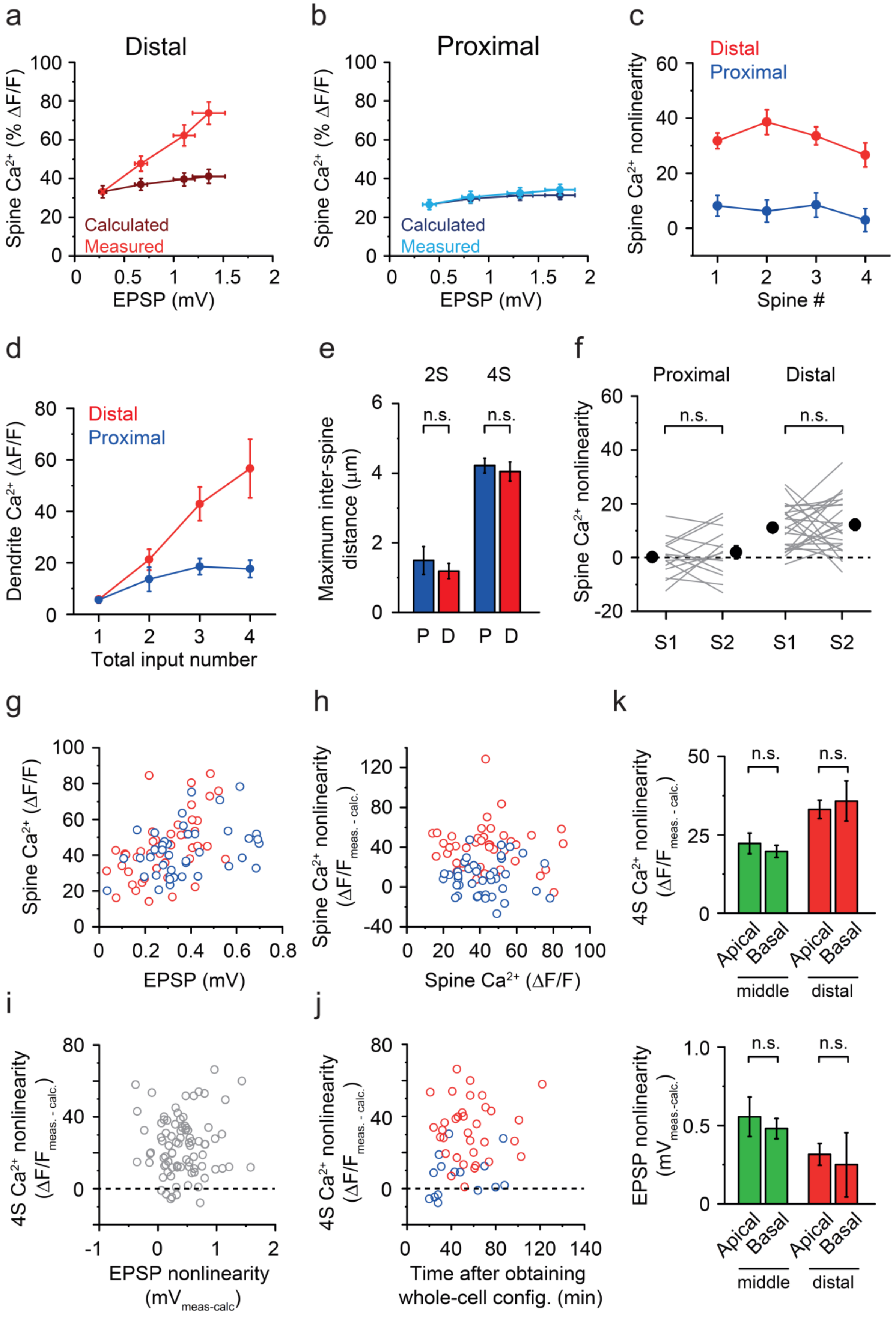
a
Distal
Spine Ca2+ (% ΔF/F)
Calculated
Measured
EPSP (mV)
b
Proximal
Spine Ca2+ (% ΔF/F)
Calculated
Measured
EPSP (mV)
c
Distal
Proximal
Spine Ca2+ nonlinearity
Spine #
d
Distal
Proximal
Dendrite Ca2+ (ΔF/F)
Total input number
e
2S
4S
n.s.
n.s.
Maximum inter-spine distance (μm)
P D P D
f
Proximal
Distal
n.s.
n.s.
Spine Ca2+ nonlinearity
S1 S2 S1 S2
g
Spine Ca2+ (ΔF/F)
EPSP (mV)
h
Spine Ca2+ nonlinearity (ΔF/Fmeas. - calc.)
Spine Ca2+ (ΔF/F)
k
4S Ca2+ nonlinearity (ΔF/Fmeas. - calc.)
n.s.
n.s.
Apical Basal Apical Basal
middle
distal
i
4S Ca2+ nonlinearity (ΔF/Fmeas. - calc.)
EPSP nonlinearity (mVmeas-calc)
j
4S Ca2+ nonlinearity (ΔF/Fmeas. - calc.)
Time after obtaining whole-cell config. (min)
EPSP nonlinearity (mVmeas-calc.)
n.s.
n.s.
Apical Basal Apical Basal
middle
distal

Supplementary Figure 2. Additional data to cooperative Ca^{2+} signaling.

(**a-b**) Calculated and measured spine Ca^{2+} signals in response to coactivation of 2-4 neighbor spines in distal (**a**, n=18 spines) and proximal (**b**, n=13 spines) dendrites (s1 and s2 pooled). Ca^{2+} nonlinearity calculated from the same dataset is shown in Fig. 1g. (**c**) Ca^{2+} nonlinearity was similar in all four spines in the set using the 4S protocol (distal: p=0.308; proximal: p=0.444, Kruskal-Wallis tests). (**d**) Dendritic Ca^{2+} signals in or near the input site. (**e**) Maximum inter-spine dendritic distance (length of dendrite stretch where the inputs were spread out) was similar for s1-s2 pairs and 4-spine sets in proximal (blue) and distal (red) dendrites (2 spines: p=0.525, 4 spines: p=0.960, Mann-Whitney tests). (**f**) Similar cooperative spine Ca^{2+} nonlinearity was measured in spine #1 and spine #2 in stimulated spine pairs (inter-spine interval: 0.1 ms) in a larger dataset both in proximal (n=14 pairs, p=0.509, Wilcoxon test) and distal (n=24 pairs, p=0.567, Wilcoxon test) dendrites. Inter-spine dendritic distance was <2.5 μm for all spine pairs. **g**) Spine Ca^{2+} signals correlated with EPSP amplitude in distal (red; Spearman R=0.544, p<0.001) and proximal (blue; Spearman R=0.328, p<0.05) spines. (**h**) No correlation between Ca^{2+} nonlinearity and initial spine Ca^{2+} signal was found either in distal (red; Spearman R=0.041, p=0.773) or in proximal (blue; Spearman R=0.018, p=0.910) spines. Proximal and distal spine data in g-h are from the same cells. (**i**) No correlation between EPSP nonlinearity and spine Ca^{2+} nonlinearity across the whole dataset with the 4S protocol (n=86, Spearman R=-0.007, p=0.942). (**j**) Spine Ca^{2+} nonlinearity using the 4S protocol was independent from the time after establishing whole-cell configuration both in distal (Spearman R=-0.030, p=0.864) and in proximal (Spearman R=0.437, p=0.102) dendritic compartments. (**k**) Spine Ca^{2+} nonlinearity (upper, data from four spines averaged) and somatic EPSP nonlinearity (lower) with the 4S protocol in middle and distal segments of apical and basal dendrites (apical, middle: n=13, distal: n=30; basal, middle: n=20, distal: n=7. Mann-Whitney tests between apical and basal dendrites: Ca^{2+} nonlinearity: middle locations p=0.645, distal locations p=0.712; EPSP nonlinearity, middle locations p=0.617, distal locations p=0.194).

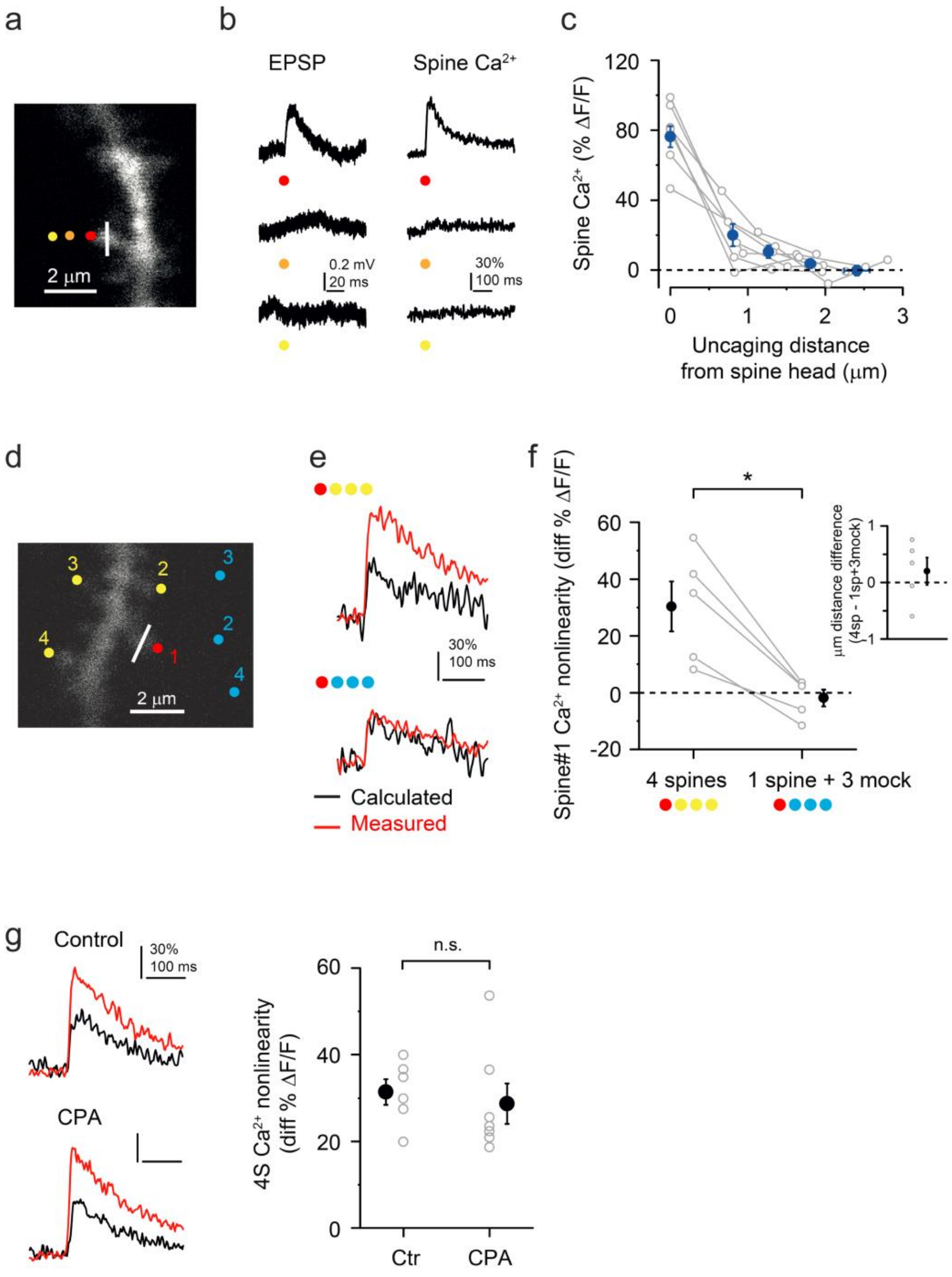

Supplementary Figure 3. Cooperative spine Ca^{2+} signaling is not due to extracellular glutamate diffusion or release from intracellular Ca^{2+} stores.

(**a**-**c**) Spatial precision of NMDAR signaling by 2P glutamate uncaging. Experiments were performed in 0.1 mM Mg^{2+} and 1 μM TTX. **a**) Single focal plane image of a distal spine with color coded uncaging locations at various distances from the spine head. (**b**) GluEPSPs and spine Ca^{2+} signals evoked by uncaging at the locations indicated in **a**. (**c**) Summary of spine Ca^{2+} signals as a function of the distance of the uncaging spot center (n=8 spines in 2 cells). Zero μm distance corresponds to uncaging spot center at the border of the spine head. (**d**-**f**) Extracellular glutamate accumulation cannot explain nonlinear spine Ca^{2+} signaling in distal dendrites. Experiments were performed in 1 mM Mg^{2+} and 1 μM TTX. (**d**) Single focal plane

image of a distal dendrite. Ca^{2+} signals in s1 (red uncaging spot) were measured alone or either with synchronous coactivation of three neighbor spines (yellow spots) or with uncaging at laterally placed uncaging spots at similar distances but >2 μm away from spines (mock uncaging, blue spots). Glutamate diffusion to s1 is expected to be similar in the two scenarios. (**e**) Cooperative spine Ca^{2+} signaling was detected in s1 only when uncaging onto neighbor spines, but not with mock uncaging in the experiment shown in **d**. (**f**) Summary data (n=5 experiments in 3 cells, $p<0.05$, Wilcoxon test). Inset: total distance of all coactive uncaging spots were similar between the 4-spine and 1-spine+3mock conditions ($p=0.500$, Wilcoxon test). (g) Left, calculated (black) and measured (red) spine Ca^{2+} signals from representative experiments using the 4S protocol (averaged data from the four spines) at distal dendritic segments under control conditions (in the presence of the vehicle DMSO, 0.03%) and after >20 min treatment with the smooth endoplasmic reticulum Ca^{2+} pump inhibitor CPA (30 μM), which depletes intracellular Ca^{2+} stores[5]. Right, summary of cooperative spine Ca^{2+} nonlinearity (n=6/7 dendrites in 3/3 control and CPA-treated cells, $p=0.353$, Mann-Whitney test).

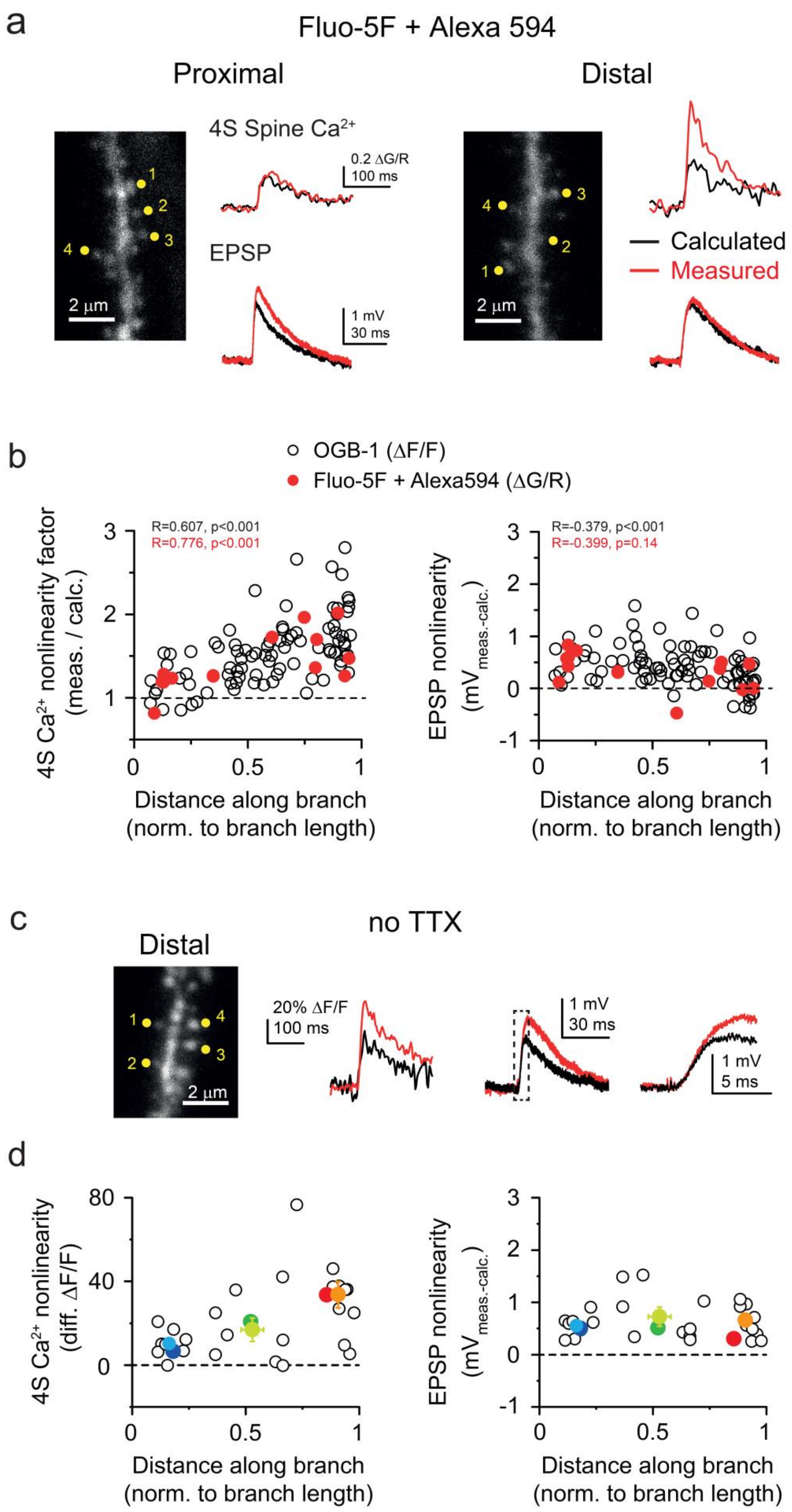

a
Fluo-5F + Alexa 594
Proximal
Distal
4S Spine Ca2+
0.2 ΔG/R
100 ms
EPSP
1 mV
30 ms
2 μm
Calculated
Measured
b
OGB-1 (ΔF/F)
Fluo-5F + Alexa594 (ΔG/R)
R=0.607, p<0.001
R=0.776, p<0.001
4S Ca2+ nonlinearity factor (meas. / calc.)
R=-0.379, p<0.001
R=-0.399, p=0.14
EPSP nonlinearity (mVmeas.-calc.)
Distance along branch (norm. to branch length)
c
no TTX
Distal
20% ΔF/F
100 ms
1 mV
30 ms
1 mV
5 ms
2 μm
d
4S Ca2+ nonlinearity (diff. ΔF/F)
EPSP nonlinearity (mVmeas.-calc.)
Distance along branch (norm. to branch length)

Supplementary Figure 4. Cooperative spine Ca^{2+} signaling does not depend on the type of Ca^{2+} indicator or the presence of TTX.

(**a**) 4S protocol (as in Fig. 2a-b) on a set of four spines in a proximal (left) and a distal (right) dendritic segment using the Ca^{2+} indicator Fluo-5F (300 μM). Changes in green Fluo-5F fluorescence were normalized to the red signal of Alexa Fluor 594 (10 μM; see Methods). (**b**) Comparison of cooperative spine Ca^{2+} signaling (left) and EPSP nonlinearity (right) measurements using OGB-1 (open black circles) and Fluo-5F (red filled circles). TTX was present in the ACSF in both experiments. Each circle represents an individual spine set (results of all four spines averaged). To directly compare results with the two dyes we used the ratio of the measured and the calculated Ca^{2+} signals. Data with OGB-1 are the same as (expressed as ΔF/F difference) in Fig. 2a. Note that some data points on the graph overlap. No difference was found between Ca^{2+} nonlinearity (two-way ANOVA: p=0.436 for dye type, p<0.001 for location, p=0.500 for interaction) or EPSP nonlinearity (two-way ANOVA: p=0.935 for dye type, p=0.039 for location, p=0.669 for interaction).

(**c**) 4S protocol on a set of four spines in a distal dendritic segment in the absence of TTX, measured using OGB-1. Right panel shows the rising phase of the EPSP, expanded from the dashed box. Note that no spikelet (as a sign of dendritic Na^{+} spike) is evident on the rising phase (compare with Fig. 6b). (**d**) Cooperative spine Ca^{2+} nonlinearity (left) and somatic EPSP nonlinearity (right) evoked by four coactivated spines at different relative locations along individual branches in the absence of TTX. Open circles represent individual spine sets (results of all four spines averaged). Filled symbols and error bars represent grouped data for proximal (relative location, RL<0.33, light blue, n=8), middle (RL: 0.33-0.67, light green, n=8) and distal (RL>0.67, orange, n=10) locations. Group data obtained in the presence of TTX (from Fig. 2a-b) are also shown for comparison (proximal: dark blue, middle: green, distal: red). No difference was found between Ca^{2+} nonlinearity (two-way ANOVA: p=0.653 for TTX treatment, p<0.001 for location, p=0.657 for interaction) whereas a slightly higher EPSP nonlinearity was observed without TTX (two-way ANOVA: p=0.045 for TTX treatment, p=0.658 for location, p=0.132 for interaction).

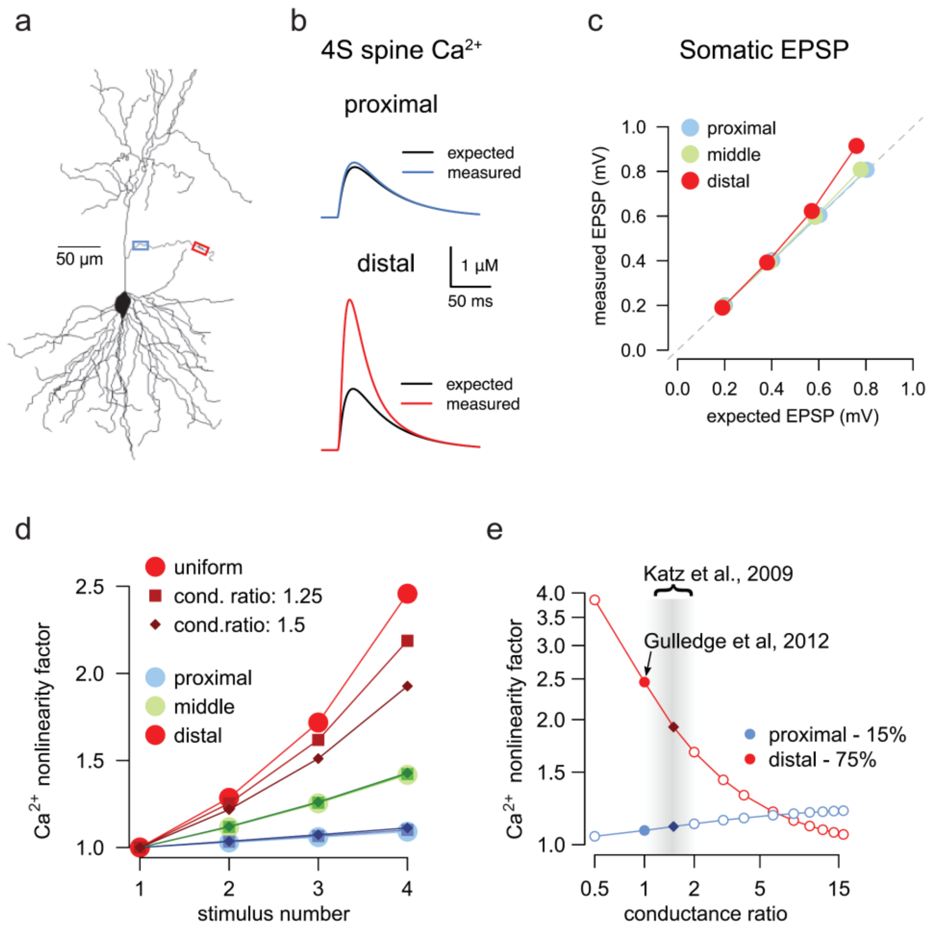

Supplementary Figure 5. Computational modelling of clustered input integration

(**a**) Image of the perisomatic dendrites of the reconstructed and modelled neuron[6], with the proximal (blue, 15% of branch length) and distal (red, 75% of branch length) stimulation sites on a branch indicated. Distal apical dendrites are not shown. (**b**) Ca^{2+} concentration change in one stimulated spine in response to the stimulation of 4 spines heads in proximal (15% of branch, blue) and distal (75% of branch, red) clusters, and the expected response based on linear summation of the responses to separate stimulations (black). Parameters were: 2 µm inter-spine distance, 0.3 ms delay. (**c**) Somatic voltage response amplitudes for 1-4 clustered input stimuli from proximal, middle and distal stimulation sites.

(**d-e**) Modelling the impact of different synaptic gradients along individual branches. We considered a range of synapse strength gradients including relatively uniform (consistent with Ref. 7-8) and proximodistally decreasing synaptic conductance (consistent with Ref. 9). (**d**) Nonlinearity of spine Ca^{2+} responses for different input locations and synaptic gradients (compare with **b**). For the simulations we used linear gradients and kept the parameters for synapses at the middle of the branch constant (hence there is no change in the integration, when the middle of the branch is stimulated, green). The gradient is quantified by the conductance ratio, that is, the ratio between the maximal conductance of the proximal and the distal synapses. The conductance ratio 1.5 between synapses at the 15% and 75% distance from the branch point is equivalent to a 2-fold reduction in the synaptic strength from the branch point to the tip, suggested by Katz et al.[9]. We applied this gradient to the maximal conductance of the AMPA

and NMDA synapses as well as to the diameter of the dendritic spine heads along the branch. (**e**) Ca^{2+} nonlinearity factor (measured response/expected response) measured for 4 inputs (as in panel **b**) as the function of the conductance ratio between proximal and distal synapses. The gradients consistent with the data of Katz et al.[9] are shown with grey shading. To achieve similar spine Ca^{2+} nonlinearity in proximal versus distal synapses a conductance ratio of ~7 would be required, i.e., distal synapses should be 7-times weaker than proximal synapses. Similar results were obtained from two other apical oblique dendrites in the modeled neuron.

Computational methods: Simulations were carried out using the NEURON simulation environment[10], using a morphologically detailed reconstruction of a CA1 pyramidal neuron[6]. The model included a membrane capacitance of 1 $\mu F/cm^2$, axial resistivity of 150 Ωcm and membrane resistivity of 20,000 Ωcm^2. Dendritic spines were modelled using a spine neck length of 1.58 μm, neck diameter of 0.077 μm, while the diameter of the spherical spine head was 0.5 μm[8]. To reproduce somatic responses to proximal and distal stimulations in the presence of the NMDA receptor blocker AP5 (data not shown), we modelled AMPA synapses as a double-exponential conductance function with rise time 0.1 ms, decay time 15 ms, reversal potential 0 mV and maximal conductance of 0.1 nS. After fixing the parameters of the AMPA receptors, we fitted the parameters of the NMDA currents to somatic responses without AP5 (not shown). The NMDA currents were modelled with double exponential kinetics using rise time 1 ms and decay time 50 ms, maximal conductance of 0.6 nS and a reversal potential 0 mV. The maximal conductances of the AMPA and NMDA receptors were set to achieve a ~ 0.2 mV depolarization at the soma in response to the stimulation of a single synapse. The voltage dependence of the NMDA receptor was modelled by multiplying the maximal conductance with a voltage dependent factor[11]: $g(V) = 1/(1 + \exp(-0.08\ V\ [Mg^{2+}] / 5))$ where the membrane potential V is measured in mV, and $[Mg^{2+}] = 1$ mM. The flow of Ca^{2+} ions through the NMDA receptors was modelled as a separate process governed by the reversal potential of the Ca^{2+} ions and the Ca^{2+} permeability of the NMDA receptors, which was set to 0.03. This was the only source of intracellular Ca^{2+} in our model. We modelled the decay of the intracellular Ca^{2+} after Graham et al. (2014)[12] which included an instantaneous buffer capacity of b=17. We set the time constant of the Ca^{2+} extrusion to 14 ms and used 0.1 μM baseline intracellular, and 2 mM extracellular Ca^{2+} concentration.

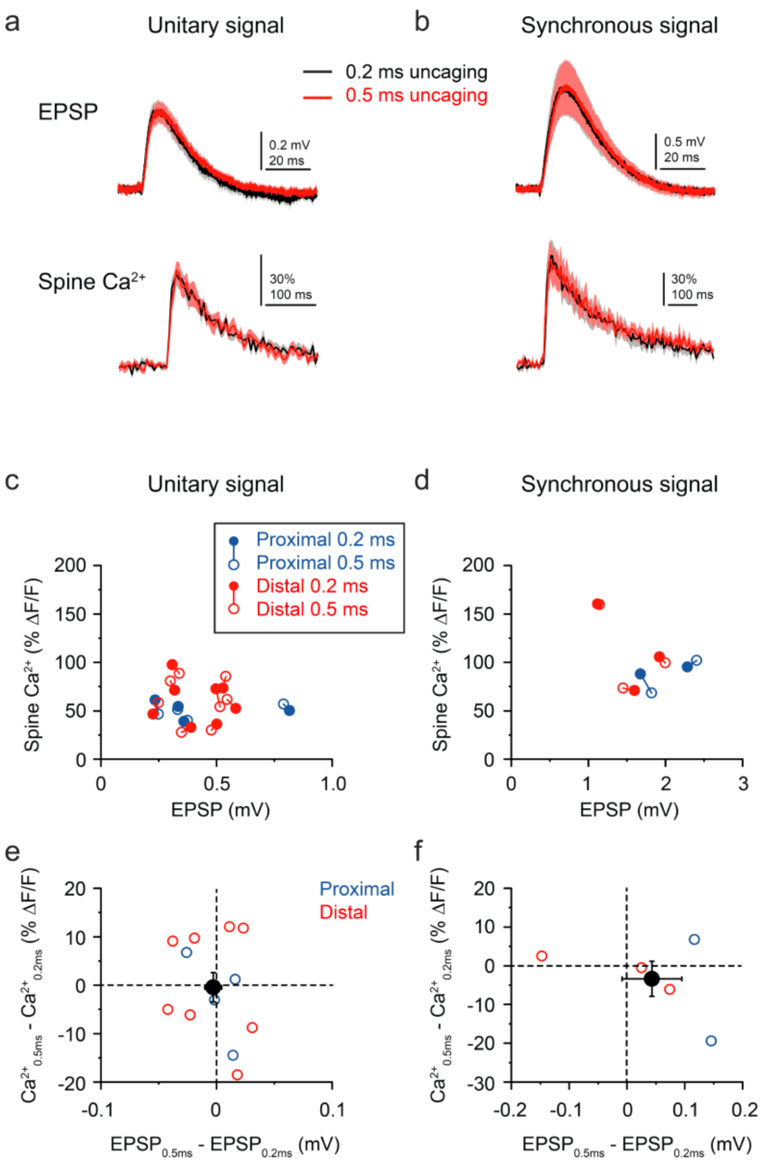

Supplementary Figure 6. Properties of EPSPs and spine Ca^{2+} signals evoked with different uncaging durations.

(**a**-**b**) Unitary (**a**) and synchronous (4 spines, **b**) uncaging-evoked gluEPSPs (upper) and spine Ca^{2+} signals (lower) using 0.2 ms (black) or 0.5 ms (red) uncaging durations at the same spines (experiments in 1 µM TTX). Uncaging laser power was lowered for the 0.5 ms uncaging duration to produce similar (<15% difference) EPSP amplitudes. Means ± SEM (shaded areas) are shown for n=12 spines with unitary and n=5 spines with synchronous recordings (data from proximal and distal spines are combined). Note the similar time course of both voltage and spine Ca^{2+} signals under the two conditions. **c**-**d**) Unitary (**c**, n=12) and synchronous (**d**, n=5) uncaging-evoked spine Ca^{2+} signals as a function of gluEPSP using 0.2 ms (filled circles) or 0.5 ms (open circles) uncaging duration in the same spines (connected circles; proximal spines in blue, distal spines in red). **e**-**f**) No difference was found in peak spine Ca^{2+} signals with different uncaging durations producing similar peak EPSP amplitudes (p=0.937 and p=0.685 for unitary signals and synchronous signals, respectively; Wilcoxon test).

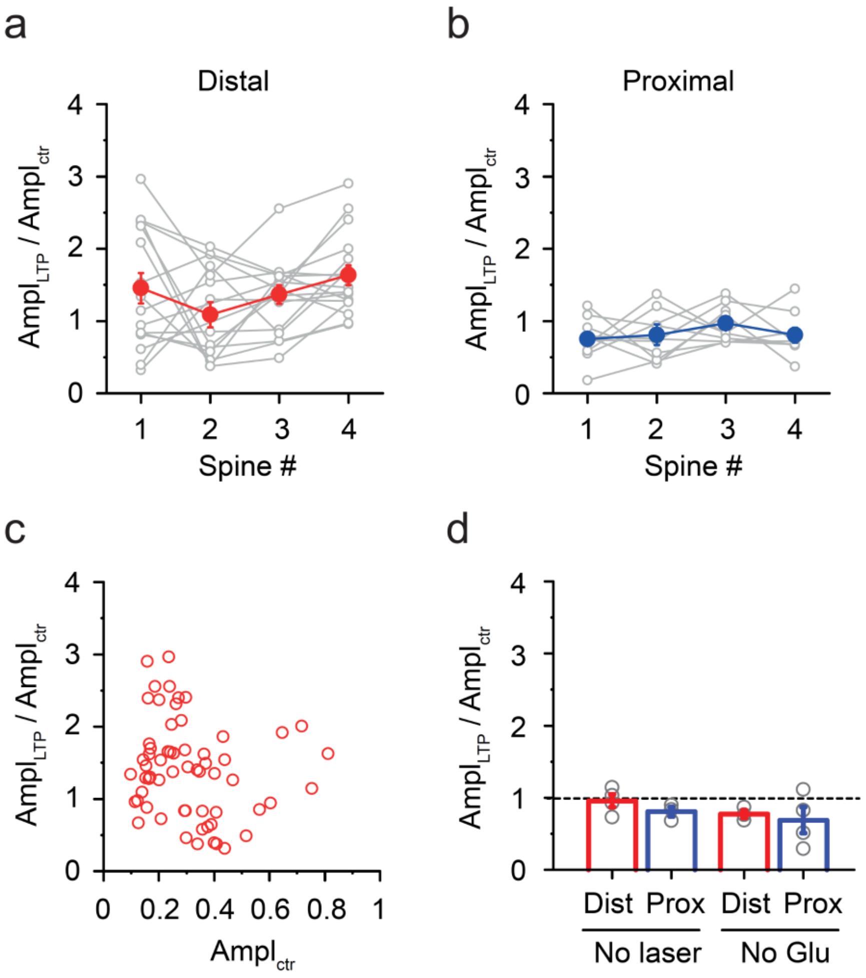

Supplementary Figure 7. Properties of cooperative LTP.

(**a-b**) Magnitude of LTP as a function of spine order in the synchronous stimulation sequence during the induction protocol for experiments conducted at distal (**c**) and proximal (**d**) dendritic segments (Friedman test p>0.05 for both). (**c**) No significant relationship between the magnitude of LTP and the initial EPSP amplitude (Spearman rank R=-0.224, p=0.081). (**d**) Summarized effect of LTP induction protocol delivered with only MNI-glutamate (‘no laser’) or only laser exposure (‘no Glu’) at distal (red; no laser: n=4 cells; no Glu: n=3 cells) and proximal (blue; no laser: n=3 cells; no Glu: n=4 cells) locations. Grey circles: averaged data from s1-4 in individual experiments.

Supplementary references

1. Nusser, Z., Naylor, D. & Mody, I. Synapse-specific contribution of the variation of transmitter concentration to the decay of inhibitory postsynaptic currents. *Biophys. J.* **80**, 1251-1261 (2001).
2. Jaffe, D.B. & Carnevale, N.T. Passive normalization of synaptic integration influenced by dendritic architecture. *J. Neurophysiol.* **82**, 3268-3285 (1999).
3. Menon, V., *et al.* Balanced synaptic impact via distance-dependent synapse distribution and complementary expression of AMPARs and NMDARs in hippocampal dendrites. *Neuron* **80**, 1451-1463 (2013).
4. Magee, J.C. & Cook, E.P. Somatic EPSP amplitude is independent of synapse location in hippocampal pyramidal neurons. *Nat. Neurosci.* **3**, 895-903 (2000).
5. Dudman, J.T., Tsay, D. & Siegelbaum, S.A. A role for synaptic inputs at distal dendrites: instructive signals for hippocampal long-term plasticity. *Neuron* **56**, 866-879 (2007).
6. Golding, N. L., Kath, W. L., & Spruston, N. Dichotomy of action-potential backpropagation in CA1 pyramidal neuron dendrites. *J. Neurophysiol*, **86**, 2998–3010 (2001).
7. Gulledge, A.T., Carnevale, N.T., & Stuart, G.J._Electrical advantages of dendritic spines. *PLoS One,* 7, e36007 (2012).
8. Harnett, M. T., Makara, J. K., Spruston, N., Kath, W. L., & Magee, J. C. Synaptic amplification by dendritic spines enhances input cooperativity. *Nature*, **491**, 599–602 (2012).
9. Katz, Y., *et al.* Synapse distribution suggests a two-stage model of dendritic integration in CA1 pyramidal neurons. *Neuron*, **63**, 171–177 (2009).
10. Hines, M. L. & Carnevale, N. T. The neuron simulation environment. *Neural Comput*., **9**, 1179–1209 (1997).
11. Major, G., Polsky, A., Denk, W., Schiller, J., & Tank, D. W. Spatiotemporally graded NMDA spike/plateau potentials in basal dendrites of neocortical pyramidal neurons. *J. Neurophysiol*, **99**, 2584–2601. (2008).
12. Graham, B. P., Saudargiene, A., & Cobb, S. Spine head calcium as a measure of summed postsynaptic activity for driving synaptic plasticity. *Neural Comput.*, **26**, 2194–2222 (2014).
